# Acute myocardial infarction and acute heart failure in the Middle East and North Africa: Study design and pilot phase study results from the PEACE MENA registry

**DOI:** 10.1371/journal.pone.0236292

**Published:** 2020-07-22

**Authors:** Khalid F. Alhabib, Habib Gamra, Wael Almahmeed, Ayman Hammoudeh, Salim Benkheddah, Mohammad Al Jarallah, Ahmed Al-Motarreb, Mothanna Alquraishi, Mohamed Sobhy, Magdi G. Yousif, Fahad Alkindi, Nadia Fellat, Mohammad I. Amin, Muhammad Ali, Ayman Al Saleh, Anhar Ullah, Faiez Zannad

**Affiliations:** 1 Department of Cardiac Sciences, King Fahad Cardiac Center, College of Medicine, King Saud University, Riyadh, Saudi Arabia; 2 Research Laboratory LR 12SP16, Fattouma Bourguiba University Hospital, University of Monastir, Monastir, Tunisia; 3 Heart and Vascular Institute, Cleveland Clinic Abu Dhabi, Abu Dhabi, United Arab Emirate; 4 Cardiology Department, Istishari Hospital, Amman, Jordan; 5 Cardiology Department, Mustapha Hospital, University Benyoucef Benkhedda, Alger Ctre, Algeria; 6 Sabah Al-Ahmad Cardia Center, Kuwait City, Kuwait; 7 Faculty of Medicine, Sana’a University, Sana’a, Yemen; 8 Ibn-Albitar Hospital for Cardiac Surgery, Baghdad, Iraq; 9 International Cardiac Center (ICC), Alexandria, Egypt; 10 Sudan Heart Centre, Khartoum, Sudan; 11 Heart Hospital, Hamad Medical Corporation, Doha, Qatar; 12 IBN SINA University Hospital, Rabat, Morocco; 13 Sh. Mohammad Bin Khalifa Cardiac Centre, Awali, Bahrain; 14 Saudi Heart Association, Riyadh, Saudi Arabia; 15 Centre d'Investigation Clinique Inserm, Institut Lorrain du Coeur et des Vaisseaux, Université de Lorraine, CHU, Nancy, France; Karolinska Institutet, SWEDEN

## Abstract

**Background:**

This pilot study describes the overall design and results of the Program for the Evaluation and Management of the Cardiac Events registry for the Middle East and North Africa (MENA) Region.

**Methods:**

This prospective, multi-center, multi-country study included patients hospitalized with acute myocardial infarction (AMI) and/or acute heart failure (AHF). We evaluated the clinical characteristics, socioeconomic and educational levels, management, in-hospital outcomes, and 30-day mortality rate of patients that were admitted to one tertiary-care center in each of 14 Arab countries in the MENA region.

**Results:**

Between 22 April and 28 August 2018, 543 AMI and 381AHF patients were enrolled from 14 Arab countries (mean age, 57±12 years, 82.5% men). Over half of the patients in both study groups had low incomes with limited health care coverage, and limited education. Nearly half of the cohort had a history of diabetes mellitus, hypertension, or hypercholesterolemia. Among patients with ST-elevation myocardial infarctions, 56.4% received primary percutaneous interventions, 24% received thrombolysis, and 19.5% received no acute reperfusion therapy. The main causes of AHF were ischemic heart diseases (55%) and primary valvular heart diseases (15%). The in-hospital and 30-day mortality rates were 2.0% and 3.5%, respectively, for AMI, and 5.4% and 7.0%, respectively, for AHF.

**Conclusions:**

This pilot study revealed a high prevalence of cardiovascular risk factors in patients with AMI and AHF in Arab countries, and low levels of socioeconomic and educational status. Future phases of the study will improve our understanding of the impact that these factors have on the management and outcomes of cardiac events in these patient populations.

## Introduction

Acute myocardial infarctions (AMI) and acute heart failure (AHF) are major causes of morbidity and mortality worldwide [1–8]. They comprise the two main reasons for emergency hospital admissions in cardiology. AMI management, including primary coronary interventions, thrombolysis, emergency care, and coronary care units (CCUs) has contributed to changing the epidemiology of coronary artery disease (CAD) in the West. Currently, those management practices are being implemented in the Middle East and North Africa (MENA) region, although with large disparities. Describing both AMI and AHF during the same time period and in the same context could facilitate future endeavors to improve management strategies.

The Arab countries in the MENA region have a population of ~350 million, which represents about 6% of the world population. These people share some common cultural, traditional, environmental, and lifestyle factors and a few gene clusters in their genomes. However, they have diverse health care systems with extremely variable national economies, ranging from low-income to high-income countries. Furthermore, most registries have been devoted to one syndrome or the other; no registry has concurrent data for AMI and AHF. These syndromes can occur concomitantly, but more frequently, they occur independently. In the last decade, several separate AMI and AHF registries were initiated in some countries of the MENA region [9–15]. However, to date, there is no single registry that includes all or most countries in the region.

This pilot study aimed to describe the overall study design of the Program for the Evaluation and mAnagement of the Cardiac Events registry for the MENA region (***PEACE MENA* Registry**) and to identify the clinical characteristics, management, in-hospital outcomes, and 30-day mortality rate of patients admitted for AMI and/or AHF in the MENA region. Moreover, for the first time in the region, we examined socioeconomic status and education levels.

## Methods

### Study design and objectives

This pilot phase is a prospective, multi-center, multi-country, cross-sectional study and included all consecutive patients hospitalized with AMI and/or AHF that underwent longitudinal follow-ups at 30 days in the out-patient clinic. The overall study objectives are:

1. to describe the demographic characteristics, clinical presentation, management, in-hospital complications, and 30-day and 1-year follow-up examinations and to identify major adverse cardiac and cerebral events (MACCE), guideline-recommended therapies, in patients with AMI and AHF in the MENA Arab countries;
2. to assess key performance indicators in the diagnostic work-up, evidence-based therapies, and cardiac procedures, at admission and during follow up, and their potential progression in Phase 2 vs. Phase 1 (see below);
3. to determine the impact of socioeconomic status (monthly household income and health care coverage) and educational levels on the management and prognosis of patients with AMI and AHF;
4. to assess well-known risk stratification scores (such as the GRACE score in AMI and the MAGGIC in AHF), and to potentially develop new risk stratification scores that might be more relevant to the MENA region.

### Study design

1. Pilot phase: This 4-month proof-of-concept phase study enrolled consecutive patients with AMI and AHF that consented to participate. In a 30-day follow-up, we recorded mortality and re-hospitalizations. We included one tertiary-care cardiac center from each country.
2. Phase 1: This 12-month recruitment phase will include consecutive patients (target = 2000 patients) with AMI and/or AHF (target: 50/50%) and conduct longitudinal follow-ups at 30 days and 1 year. Patients will be recruited from hospitals with and without catheterization laboratories to reflect real-life health care conditions. The target number of patients in each country was arbitrarily estimated based on recruitment feasibility, in view of the current economic and national security challenges in the Arab countries.
3. Phase-2: This phase will repeat phase 1, but 6 months later. This phase aims to assess changes in pre-defined key performance indicators and outcomes,. The target number of patients is 2000 (AMI/AHF: 50%/50%).

### Inclusion criteria

1. Patients hospitalized with Type 1 AMI, including patients with ST-elevation myocardial infarctions (STEMI) and non-STEMI (NSTEMI).
2. Patients hospitalized with AHF.
3. Age ≥18 years
4. Written informed consent.

AMI and AHF were defined according the European Society of Cardiology guidelines [16, 17].

### Exclusion criteria

1. Patients unwilling to provide written informed consent;
2. AMI due to an imbalance in oxygen supply and demand; AMIs likely to result in death with no available biomarkers; peri-procedural AMIs secondary to a percutaneous coronary intervention (PCI) or coronary artery bypass surgery (CABG); i.e., AMI Types 2−5.

### Data collection

1 A standardized on-line case report form (S1 File) was used to record data prospectively at hospital admission (www.peace-mena-registry.org). Baseline data: demographic characteristics, socioeconomic strata, health care barriers, educational levels, cardiovascular risk factors, clinical presentation, laboratory investigations, including cardiac troponin and N-terminal pro-B type natriuretic peptide (NT-proBNP) collected at hospital admission, cardiac procedures, and treatments;
2 Management and follow-up data: In-hospital course, cardiac procedures, medical therapy at discharge, and clinical outcomes, collected at discharge, 30-days (all phases), and 1-year post-discharge (Phase 1 and Phase 2) follow up data are collected from on-line case report forms (S1 File). MACCE was defined as the composite of death, non-fatal stroke, non-fatal recurrent MI, emergency revascularization for acute ischemia or MI, and re-hospitalization for AHF that required intravenous diuretic treatment.

#### Key performance indicators

We will compare phase 2 to phase 1 data on the following key performance indicators:

3 AMI group: use of high-sensitivity cardiac troponin upon admission (vs. the regular cardiac troponin), echocardiography, oral evidence-based treatments at hospital discharge (i.e., anti-platelets, statins, beta-blockers, angiotensin converting enzyme inhibitors (ACE-Is), or angiotensin receptor blockers (ARBs), door-to-needle time and door-to-wire/balloon times (for STEMI), coronary catheterization, and revascularization with PCI or CABG.
4 AHF group: NT-proBNP measured upon admission, echocardiography, and oral HF treatments at hospital discharge and during a long-term follow-up (i.e., beta-blockers, ACE-Is, ARBs, aldosterone antagonist, angiotensin receptor neprilysin inhibitor (ARNI), implantable cardioverter-defibrillator, and cardiac resynchronization therapy device).

### Study coordination

The overall study was coordinated and data were monitored for completeness and correctness by the Principal Investigator (Prof. Khalid F Alhabib) and a research coordinator dedicated to the study (Muhammad Ali) at the Saudi Heart Association (Riyadh, Saudi Arabia).

### Statistical analysis

Data are expressed as the means ± standard deviation (SD) for normal distributions and the median and interquartile range (IQR) for non-normal distributions. Differences between groups were assessed with the chi square or Fisher’s exact test for categorical variables. The student t-test was used for continuous, normally distributed variables, and the Mann–Whitney U test was used for skewed variables. P-values<0.05 were considered significant. All analyses were performed with SPSS software.

### Ethics statement

Ethics approval was obtained from the Institutional Review Boards (IRBs) of the participating countries according to their relevant national regulations and laws. Saudi Arabia, King Saud University, College of Medicine IRB, OHRP No. IORG0006829. Qatar, Doha, HMC-IRB Registration: SCH-HMC-020-2015. Algeria, Hopital Mustapha Place du Premier Mai- Alger IRB. Kuwait, Ministry of Health, Secretary for Planning and Quality Committee. Yemen, Al Thawra Hospital Ethics Committee. Iraq, Baghdad Health Department, Ibn Naphis Hospital IRB. Egypt, Alexandria, International Cardiac Center IRB. Tunisia, Ministry of Health, Sahloul Hospital of Sousse. UAE, Hammoud Hospital IRB. Sudan, Sudan Heart Center IRB. Jordan, Istishari Hospital IRB. Morocco, Centre Hospitalo–Universitaire Ibn Sina IRB. Bahrain, BDF Royal Medical Services IRB. Oman, Ministry of Health, Directorate General of Planning and Studies. written consent form was obtained.

## Results

**Fig 1:**
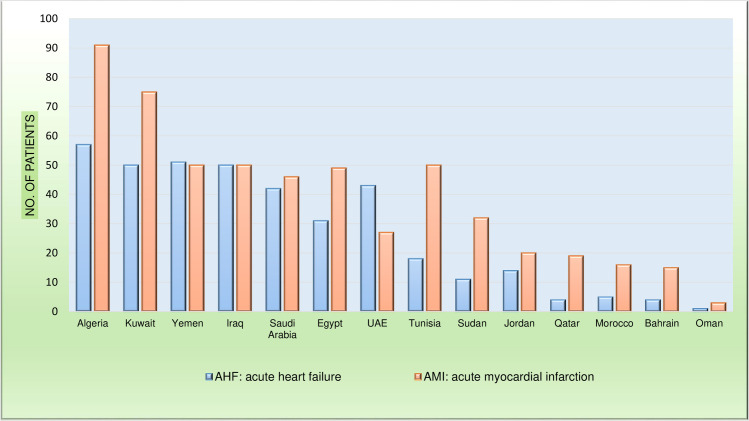
Numbers of patients with AMI and AHF enrolled from each country.

Between 22 April and 28 August 2018, 543 patients with ACS and 381 patients with AHF were enrolled from 14 Arab countries (Fig 1).

### Acute myocardial infarction

Among patients with AMI (mean age ± SD), 57±12 years, 82.5% males), 312 (57%) had STEMI and 231 (43%) had NSTEMI. The median (IQR) monthly income was 600 (700) US$, and households included a median of five family members. Just over half of the patients received governmental medical care, and 57% had difficulty affording additional medical expenses. Unemployment was as high as 18.8%, and 20% was retired. Over one-third had no or only primary school education, and 3.6% had advanced education (e.g., PHD, master’s degree equivalent; (S1 Table). Furthermore, compared to AMI patients with higher income (>500 US$/month), those with lower income (≤500 US$/month) were more likely to have low education, have history of angina, MI, orstroke, present with STEMI, and were less likely to receive timely primary PCI for patients with STEMI (door-to-balloon time <90 minutes), while those with higher income were more likely to be males and to be from the Gulf Cooperation Council (GCC) countries (all p values are significant, S2 Table).

The prevalence of cardiovascular risk factors was high; 53.6% had diabetes mellitus, 55% had hypertension, 43.6% had hypercholesterolemia, and 55.6% were either current or ex-smokers. Upon presentation, 22.5% were transferred to the emergency department in a hospital ambulance and 8.3% were transferred by the Emergency Medical Service (EMS). Compared to patients with STEMI, those with NSTEMI were more likely to have history of angina, myocardial infarction, PCI, CABG, heart failure, chronic renal failure, diabetes, hypertension, or hypercholesterolemia (Table 1). The overall rate of HF histories was 5.9%, and HF Killip classifications upon admission were not significantly different between patients with STEMI and those with NSTEMI.

**Table 1:** Baseline characteristics of the AMI population.

Characteristic	Total N = 543	STEMI N = 312 (57.46%)	NSTEMI N = 231 (42.54%)	P-value
Age (y), mean ±SD	57.45 ±12.01	56.18 ±11.46	59.17 ±12.54	0.004
Male	448 (82.50%)	258 (82.69%)	190 (82.25%)	0.894
**Medical History**				
Angina	171 (31.49%)	78 (25.00%)	93 (40.26%)	< .001
MI	108 (19.89%)	32 (10.26%)	76 (32.90%)	< .001
PCI	87 (16.02%)	30 (9.62%)	57 (24.68%)	< .001
CABG	20 (3.68%)	4 (1.28%)	16 (6.93%)	< .001
Heart failure	32 (5.89%)	13 (4.17%)	19 (8.23%)	0.047
Stroke	30 (5.52%)	13 (4.17%)	17 (7.36%)	0.107
Chronic renal failure	22 (4.05%)	8 (2.56%)	14 (6.06%)	0.041
BMI (kg/m^2^), mean ±SD	27.34±4.60	26.96±4.04	27.85±5.24	0.025
Diabetes	291 (53.59%)	156 (50.00%)	135 (58.44%)	0.051
HTN	297 (54.70%)	149 (47.76%)	148 (64.07%)	<0.001
Hypercholesterolemia	237 (43.65%)	124 (39.74%)	113 (48.92%)	0.033
Current or ex-smoker	302 (55.62%)	183 (58.65%)	119 (51.52%)	0.098
**Transfer Modalities**				
Transfer by ambulance	122 (22.47%)	83 (26.60%)	39 (16.88%)	0.007
Transfer by Emergency Medical Service (Red Crescent or Red Cross)	45 (8.28%)	29 (9.29%)	16 (6.92%%)	0.322
**Vital Signs on admission**				
HR>100 bpm	64 (11.79%)	44 (14.10%)	20 (8.66%)	0.052
SBP<90 mm Hg	12 (2.21%)	11 (3.53%)	1 (0.43%)	0.015
**CHF Killip Class**				
Class I	450 (82.87%)	252 (80.77%)	198 (85.71%)	0.260
Class II	64 (11.79%)	43 (13.78%)	21 (9.09%)	
Class III (Pulmonary edema)	24 (4.42%)	13 (4.17%)	11 (4.76%)	
Class IV (Cardiogenic Shock)	5 (0.92%)	4 (1.28%)	1 (0.43%)	
**Cardiac biomarkers available**				
cTn	200 (36.83%)	128 (41.03%)	72 (31.17%)	0.019
hs-cTn	343 (63.17%)	184 (58.97%)	159 (68.83%)	
BNP	79 (14.5%)	41 (13.14%)	38 (16%)	0.279
Value) pg/ml), Median (IQR)	319 (1455)	80 (390)	857 (2851)	<0.001
NT-proBNP	45 (36.29%)	21 (33.87%)	24 (38.71%)	
value) pg/mL(, Median (IQR)	442 (3197)	568 (5414)	397 (1889)	<0.001
**Echocardiography parameters**				<0.001
Normal LVSD (EF>50%)	209 (43.91%)	90 (34.88%)	119 (54.59%)	
Mild LVSD (EF: 40–50%)	169 (35.50%)	107 (41.47%)	62 (28.44%)	
Moderate LVSD (EF: 30–40%)	72 (15.13%)	46 (17.83%)	26 (11.93%)	
Severe LVSD (EF<30%)	26 (5.46%)	15 (5.81%)	11 (5.05%)	
Elective coronary angiogram	198 (36.46%)	73 (23.40%)	125 (54.11%)	<0.001
Elective PCI	119 (21.91%)	47 (15.06%)	72 (31.11%)	<0.001
CABG	29 (5.34%)	8 (2.56%)	21 (9.09%)	<0.001

Values are the number of patients (%), unless indicated otherwise. MI: myocardial infarction, AMI: acute MI, PCI: Percutaneous coronary intervention, CABG: Coronary artery bypass surgery, BMI: Body mass index, HTN: hypertension, HR: Heart Rate, SBP: systolic blood pressure, CHF: chronic heart failure; cTn: Cardiac troponin, hs-cTn: High sensitivity cardiac troponin, BNP: B-type natriuretic peptide, NT-proBNP: N-terminal Pro B-type natriuretic peptide, LVSD: left ventricular systolic dysfunction, EF: ejection fraction.

**Fig 2:**
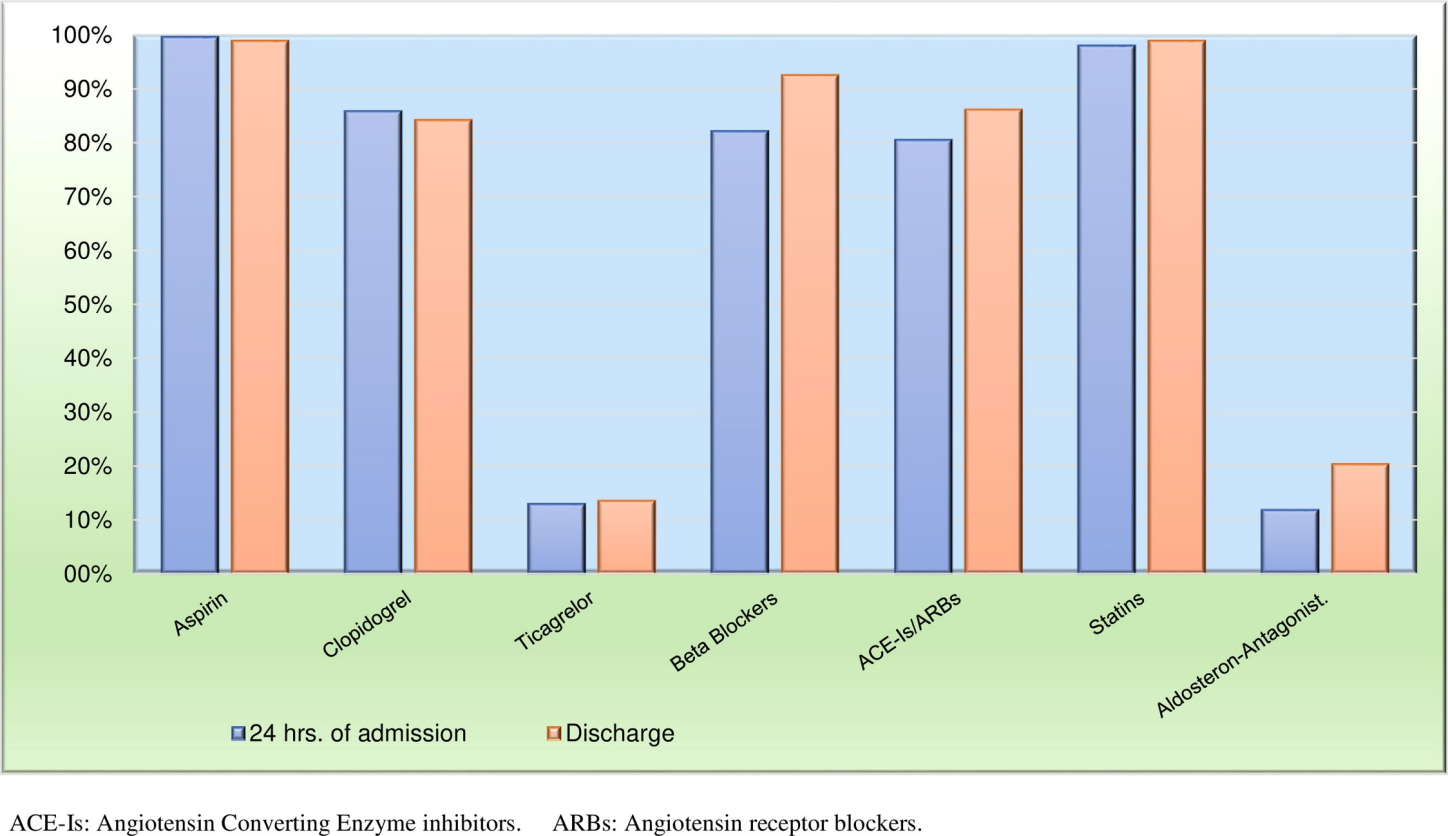
Medications given within 24 hrs. of hospital admission and at hospital discharge in the AMI group. AMI: acute myocardial infarction.

In the STEMI group, 56.4% of patients received a primary PCI, 24% received thrombolysis, and 19.5% received no acute reperfusion therapy. Door-to-balloon times<90 min were achieved in nearly two thirds of patients (S3 Table). Guideline-recommended treatments were given at high rates upon hospital admission: 99.6% of patients received acetylsalicylic acid (ASA), 98% received statins, 82% received beta-blockers, and 80.5% received ACE-I/ARBs. Interestingly, only 12.9% received Ticagrelor, compared to 85.8% that received Clopidogrel (Fig 2). The overall use of guideline-recommended treatments remained high upon discharge: 98% received ASA, 92% received beta-blockers, 86% received ACE-I/ARBs, and 98% received statins. The use of Ticagrelor increased, overall, to 16% at hospital discharge, and it was given significantly more frequently to patients with STEMI (22%) compared to those with NSTEMI (8%, p<0.001).

In-hospital complications are shown in Table 2. In-hospital and 30-day mortality rates were 2.0% and 3.5%, respectively. Furthermore, compared to AMI patients with higher income (>500 US$/month), those with lower income (≤500 US$/month)were more likely to have in-hospital recurrent ischemia (12.1% vs. 5.9%, p value = 0.025) and cardiogenic shock (5.6% vs. 1.8%, p value = 0.039).

**Table 2:** In-hospital outcomes and 30-day mortality in the AMI population.

Outcomes	Total N = 543 (%)
Recurrent ischemia	38 (7.00%)
Recurrent MI	5 (0.92%)
AF or flutter	25 (4.60%)
HF	82 (15.10%)
Cardiogenic Shock	22 (4.05%)
VF or VT cardiac arrest	34 (6.26%)
Intra-aortic balloon pump	1 (0.18%)
Stroke	6 (1.10%)
Major bleeding	2 (0.37%)
Cardiac tamponade	1 (0.18%)
Stent thrombosis	5 (0.92%)
In-hospital mortality	11 (2.03%)
One-month mortality	15 (3.54%)

MI: Myocardial Infarction, AMI: acute MI; AF: Atrial Fibrillation, HF: Heart Failure, VF: Ventricular fibrillation, VT: Ventricular Tachycardia

### Acute heart failure

The AHF group (mean age, 60.6±15.4 years; 67% males) included 237 (62.2%) patients with acute on chronic HF and 144 (37.8%) patients with acute de novo HF. More than half of the AHF population earned less than 500 US$ monthly, and approximately one third earned 500–2000 US$. Households included a median of four family members. Governmental medical care was provided to 43%, and 60% had difficulty affording additional medical expenses. Unemployment was as high as 39%, and one third was retired. Over half of the patients had no or only primary school education, and 2.1% had an advanced education (S4 Table). Furthermore, compared to AHF patients with higher income (>500 US$/month), those with lower income (≤500 US$/month)were more likely to be younger and to present with de novo HF, while those with higher income were more likely to be from the GCC countries, have higher rates of CAD risk factors, history of angina or MI, and ischemic heart disease as the main cause of HF (S5 Table).

Compared to patients with de novo AHF, patients with acute on chronic HF were more likely to have a history of CAD, valvular heart disease, atrial fibrillation, chronic kidney disease, dialysis, or hyperlipidemia (Table 3). Tachycardia and CCU/ICU admissions occurred more frequently with acute de novo HF (57.6%) compared to acute on chronic HF (44.3%, p<0.001). Upon presentation, 18% were transferred to the emergency department in an ambulance. The overall rate of HF with preserved ejection fraction (HFpEF, defined as ≥50% left ventricular ejection fraction) was 20.8%. Significantly more patients had HFpEF in the acute de novo HF group (26%) than in the acute on chronic HF group (17%; p = 0.005).

**Table 3:** Baseline characteristics of the AHF population.

Characteristic	Total N = 381	Acute on Chronic HF N = 237 (62.20%)	Acute De novo HF N = 144 (37.80%)	P-value
Age (y), mean±SD	60.61±15.37	62.11±14.07	58.15±17.05	0.015
Male	255 (66.93%)	160 (67.51%)	95 (65.97%)	0.757
**Admission Location**				
CCU or ICU	188 (49.34%)	105 (44.30%)	83 (57.64%)	<0.001
Monitored Bed	105 (27.56%)	84 (35.44%)	21 (14.58%)	
Unmonitored Bed	88 (23.10%)	48 (20.25%)	40 (27.78%)	
**Medical History**				
CAD (angina or myocardial infarction)	196 (51.44%)	145 (61.18%)	51 (35.42%)	<0.001
HF	236 (61.94%)	236 (99.58%)	0 (0.00%)	<0.001
PCI	77 (39.29%)	56 (38.62%)	21 (41.18%)	0.748
CABG	33 (16.84%)	30 (20.69%)	3 (5.88%)	0.015
VHD	72 (18.90%)	59 (24.89%)	13 (9.03%)	<0.001
CHD	5 (1.31%)	3 (1.27%)	2 (1.39%)	0.918
AF	85 (22.31%)	66 (27.85%)	19 (13.19%)	<0.001
PAD	12 (3.15%)	10 (4.22%)	2 (1.39%)	0.125
Stroke	35 (9.19%)	25 (10.55%)	10 (6.94%)	0.238
Diabetes	204 (53.54%)	138 (58.23%)	66 (45.83%)	0.019
Hypertension	242 (63.52%)	156 (65.82%)	86 (59.72%)	0.230
CKD	88 (23.10%)	75 (31.65%)	13 (9.03%)	<0.001
Dyslipidemia	151 (39.63%)	106 (44.73%)	45 (31.25%)	0.009
Current Smoking	91 (23.88%)	38 (16.03%)	53 (36.81%)	<0.001
**Ambulance**	69 (18.11%)	40 (16.88%)	29 (20.14%)	
**Vital Signs on admission**				
HR>100 bpm	125 (32.81%)	62 (26.16%)	63 (43.75%)	<0.001
SBP<90 mmHg	31 (8.14%)	18 (7.59%)	13 (9.03%)	0.620
**Cardiac biomarkers**				
***Troponin I / T***				0.025
Not Done	88 (23.72%)	60 (26.32%)	28 (19.58%)	
Elevated	137 (36.93%)	72 (31.58%)	65 (45.45%)	
Normal	146 (39.35%)	96 (42.11%)	50 (34.97%)	
***Troponin Type***				<0.001
cTn available	122 (43.88%)	56 (33.53%)	66 (59.46%)	
Hs-cTn available	156 (56.12%)	111 (66.47%)	45 (40.54%)	
***BNP/NT-ProBNP***				
BNP/NT-ProBNP available	41 (16.40%)	31 (18.79%)	10 (11.76%)	0.155
BNP (pg/ml)	306.9±297.1	313.5±323.7	282.7±188.7	0.826
NT-ProBNP (pg/ml)	8551±13107	9869±14291	3362±3640	0.076
**Echocardiography parameters**				0.005
Normal LV function (EF>50%).	76 (20.49%)	39 (17.03%)	37 (26.06%)	
Mild LV dysfunction (EF = 40–50%)	84 (22.64%)	45 (19.65%)	39 (27.46%)	
Moderate LV dysfunction (EF = 30–39%).	91 (24.53%)	57 (24.89%)	34 (23.94%)	
Severe LV dysfunction (EF<30%)	120 (32.35%)	88 (38.43%)	32 (22.54%)	
**Angiographic parameters**				
Coronary Angiogram	92 (24.15%)	40 (16.88%)	52 (36.11%)	<0.001
PCI	44 (11.55%)	17 (7.17%)	27 (18.75%)	<0.001
CABG	4 (1.05%)	0 (0.00%)	4 (2.78%)	0.010

Values are the number of patients (%), unless indicated otherwise. AHF: acute heart failure; CCU: Coronary Care Unit, ICU: Intensive Care Unit, CAD: Coronary artery disease, HF: Heart Failure, PCI: Percutaneous coronary intervention, CABG: Coronary artery bypass surgery, VHD: Valvular Heart Disease, CHD: Congenital Heart Disease, AF: Atrial Fibrillation, PAD: Peripheral arterial disease, CKD: Chronic kidney disease, cTn: Cardiac Troponin, Hs-cTn: High Sensitivity Cardiac Troponin, BNP: B-type natriuretic peptide; NT-proBNP: N-terminal prohormone-BNP, LV: left ventricular

Serum cardiac troponin was significantly more elevated in the acute de novo HF group (59.4%) compared to the acute on chronic HF group (33.5%, p<0.001). Brain natriuretic peptide (BNP) and NT-proBNP were detected at an overall rate of 16.4%. The main causes of HF were ischemic heart disease (55%) and primary valve disease (15%). Furthermore, the most common precipitating factors of AHF were AMI (21%), infection (18.5%), and noncompliance with medications (14.6%; Table 4).

**Table 4:** Main causes and main precipitating factors of the AHF population.

Main causes of heart failure	Total N = 381 (%)
Ischemic heart disease	210 (55.26%)
Primary valve disease	57 (15.00%)
Hypertensive heart disease	29 (7.63%)
Hypertrophic cardiomyopathy	6 (1.58%)
Myocarditis	3 (0.79%)
Idiopathic cardiomyopathy	27 (7.11%)
Cardiotoxic cardiomyopathy	2 (0.53%)
Right-sided heart failure	26 (6.84%)
Pregnancy-related cardiomyopathy	6 (1.58%)
Others	14 (3.68%)
**Main precipitating factors of acute heart failure**	
Acute myocardial infarction	80 (21.16%)
Infection	70 (18.52%)
Noncompliance with medications	55 (14.55%)
Others	47 (12.43%)
Uncontrolled arrhythmias	37 (9.79%)
Worsening renal failure	30 (7.94%)
Noncompliance with diet	22 (5.82%)
Uncontrolled hypertension	20 (5.29%)
Anemia	16 (4.23%)
Unknown	1 (0.26%)

**Fig 3:**
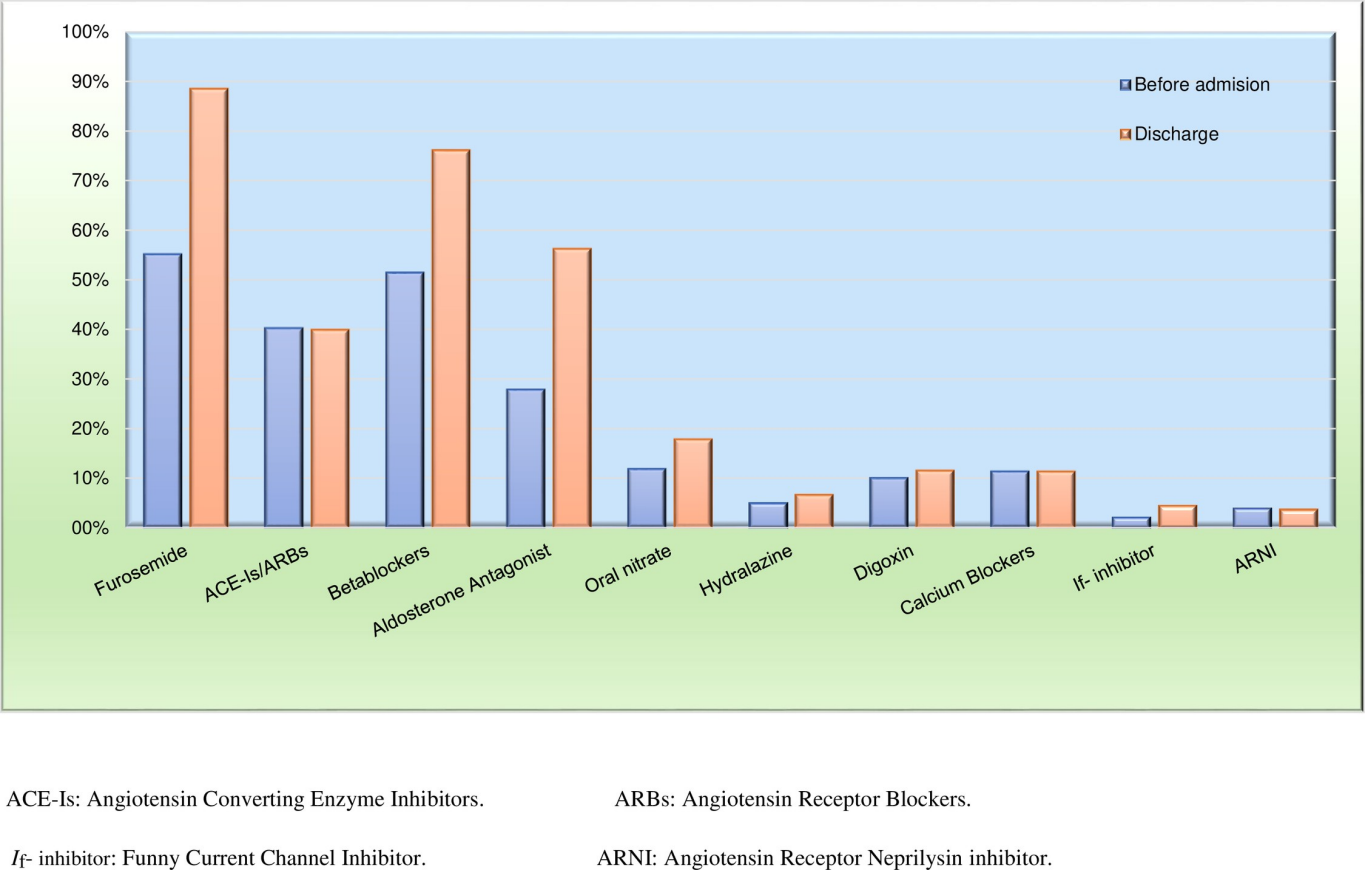
Medications given before hospital admission and at hospital discharge in the AHF group. AHF: acute heart failure.

At hospital discharge, 88.5% of patients were taking furosemide, 76.1% were taking betablockers, 39.9% were taking renin-angiotensin system inhibitors, 56.2% were taking aldosterone antagonist, and 3.7% were taking ARNI (Fig 3). The in-hospital and 30-day mortality rates were 5.4% and 7.0%, respectively (Table 5). There were no significant differences in the hard cardiovascular outcomes between AHF patients with lower versus higher income (S5 Table).

**Table 5:** In-hospital outcomes and 30-day mortality in the AHF population.

Events	Total N = 381 (%)
Intubation/ Ventilation	31 (8.14%)
Intra-aortic balloon pump	16 (4.20%)
Acute Dialysis/ Ultrafiltration	13 (3.41%)
VF or VT cardiac arrest	27 (7.09%)
AF or flutter	40 (10.50%)
Major bleeding	4 (1.05%)
Blood transfusion	13 (3.41%)
Stroke	3 (0.79%)
Systemic infection that requires antibiotics	89 (23.36%)
In-hospital mortality	20 (5.41%)
One-month mortality	25 (7.08%)

VF: Ventricular fibrillation, VT: Ventricular tachycardia, AF: Atrial fibrillation

## Discussion

The PEACE MENA registry was the first registry to include a majority of Arab countries in the MENA regions. It has provided data on the current management of AMI and AHF, described with standardized definitions and data variables. The present study assessed–for the first time–the socioeconomic and educational status of these patient populations.

The main findings of this pilot-phase study of the PEACE MENA registry were that patients in the MENA region were relatively young at clinical presentation and had a high prevalence of cardiovascular risk factors, compared to patients in Western countries. Similarly, previous registry studies conducted in the region reported a high prevalence of cardiovascular risk factors [9–15]. This high risk profile highlighted the urgent need of preventive measures, screening for early detection, and adequate control of cardiovascular risk factors. The remarkably low 2% in-hospital mortality rate found in this pilot phase study was likely due to the high acute reperfusion rate (exceeding 80%) performed by the enrolled tertiary-care centers.

The distribution of ACS types in the PEACE MENA cohort was quite different from the distributions described in previous registries. We found a higher prevalence of STEMI (57%) than NSTEMI (43%). These prevalences were 46% and 54% in ACCESS [14], 39% and 61% in Gulf RACE [10], and 41.5% and 68.5% in SPACE [9] registries. However, our findings were similar to those reported recently in the STARS registry of Saudi Arabia (65% vs. 35%, respectively) [15]. This discrepancy might be explained by the fact that the majority of centers that participated in the pilot phase of the PEACE MENA were major tertiary care centers with catheterization laboratory facilities that offered primary PCI service; thus, more patients with STEMI were recruited. In addition, previous ACS registries in our region included patients with unstable angina, which increased the overall proportion of patients with NSTEACS, compared to the proportion of patients with STEMI. Another interesting finding was the strikingly low rate of patients with STEMI that were transferred to the emergency department in a hospital ambulance (22.5%) or by the EMS (8.3%). These findings were comparable to findings in the Gulf RACE 3Ps registry [12]. In the French Fast MI registry, 62% of patients with STEMI were transported by the EMS to the hospital, which might have contributed to the low in-hospital mortality rate (2.7%) in that population [18, 19]. However, they are large global variations in the use of reperfusion therapies, ranging from 53.9% in India up to 81.2% in Southern Europe [20].

In the AHF population, the main cause of HF was ischemic heart diseases (55%). This finding reflected the epidemiological transition witnessed in the region over the last few decades, due to the high prevalence of CAD risk factors, and substantial reductions in rheumatic and valvular heart diseases. The overall HFpEF rate was ~20%, compared to 30–50% reported in other international registries [21]. This result was consistent with our previous report on the Heart Function Assessment Registry Trial in Saudi Arabia [13]. That study showed a high prevalence of ischemic heart diseases, and hence, a high proportion of myocardial injury and subsequent HF, with reduced ejection fractions, rather than HFpEF. The in-hospital mortality for the AHF group was just over 5%, similar to previous reports from the region [13], but relatively high, compared to the ~3% mortality reported in Western populations [22]. Notably, and consistent with previous international reports, the post-AHF event rate was larger (double, herein) than the post-AMI event rate. However, unlike our study, the majority of international registry studies collected AHF and AMI data separately (i.e., not at the same sites, during the same time period).

Regarding the socioeconomic status of patients with AMI and AHF, over half of the patients in both study groups had relatively low incomes, the majority had limited educations, and there were more in-hospital complications in the AMI group with lower income. In the Prospective Urban Rural Epidemiological study, the major cardiovascular event rates were higher in middle- and low-income countries than in high-income countries, despite a lower risk-factor burden in low-middle income countries [23]. Furthermore, patients with AHF from low-middle income countries with large socioeconomic inequalities had relatively high mortality rates and poor HF outcomes [24, 25].

This study had some limitations, including those inherent to observational studies, such as potential selection bias and missing or incomplete information. Participation was voluntary, and although enrolling consecutive patients was encouraged, it was not verified, similar to other registry studies. Future phases of the study will further assess the impact of socioeconomic and educational status on the clinical outcomes of these patient populations.

## Supporting information

S1 File AMI and AHF Case Report forms. (DOCX)

S1 Table Educational level and socio-economic status in the ACS population. (DOCX)

S2 Table Clinical features, management, and outcomes of patients with lower versus higher income presenting with acute myocardial infarction. (DOCX)

S3 Table Modalities and timing of reperfusion in the STEMI population. (DOCX)

S4 Table Educational level and socio-economic status in the AHF population. (DOCX)

S5 Table Clinical features, management, and outcomes of patients with lower versus higher income presenting with acute heart failure. (DOCX)
