# Correction: Acute Myocardial Infarction and Acute Heart Failure in the Middle East and North Africa: Study Design and Pilot Phase Study Results from the PEACE MENA Registry

**DOI:** 10.1371/journal.pone.0246036

**Published:** 2021-01-27

**Authors:** Khalid F. Alhabib, Habib Gamra, Wael Almahmeed, Ayman Hammoudeh, Salim Benkhedda, Mohammad Al Jarallah, Ahmed Al-Motarreb, Mothanna Alquraishi, Mohamed Sobhy, Magdi G. Yousif, Fahad Alkindi, Nadia Fellat, Mohammad I. Amin, Muhammad Ali, Ayman Al Saleh, Anhar Ullah, Faiez Zannad

The fifth author's name is spelled incorrectly. The correct name is: Salim Benkhedda. The correct citation is: Alhabib KF, Gamra H, Almahmeed W, Hammoudeh A, Benkhedda S, Al Jarallah M, et al. (2020) Acute myocardial infarction and acute heart failure in the Middle East and North Africa: Study design and pilot phase study results from the PEACE MENA registry. PLoS ONE 15(7): e0236292. https://doi.org/10.1371/journal.pone.0236292

There is also an error in affiliation 5 for author Salim Benkhedda. The correct affiliation 5 is: Cardiology Oncology Collaborative Research Group, Faculty of Medicine, Benkhedda Benyoucef University, Algeria.

A research assistant’s name is also spelled incorrectly in the Acknowledgements section. The correct name is: Dahlia Djermane.
