# Supplementary material for: Acute myocardial infarction and acute heart failure in the Middle East and North Africa: Study design and pilot phase study results from the PEACE MENA registry
Source: PLoS One. 2020 Jul 22;15(7):e0236292. doi: 10.1371/journal.pone.0236292 (PMC7375595; doi:10.1371/journal.pone.0236292)
Supplement: S1 File — (DOCX) [file pone.0236292.s001.docx]

**S 1 File.
AMI and AHF Case Report forms**

**AMI, CRF of Cath-lab hospitals** **
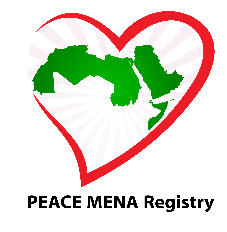
**

Part-1 Demographics

INCLUDE:

Consecutive patients hospitalized with Type 1 AMI (STEMI and NSTEMI) defined according to the ESC Guidelines, Age ≥ 18 years of age, written informed consent

EXCLUDE:

AMIs due to imbalance of oxygen supply and demand, AMIs resulting in deaths but without availability of biomarkers, and AMIs related to PCI or CABG (i.e.: AMI Types 2, 3, 4, and 5; respectively)

* denotes optional items

PT. registry ID: automatically generated by the system when you fill the data from health care sector to ethnicity and click save. Write it back after you enroll the case

*****MRN:

Geographical. Region: 🞆Riyadh 🞆Qassim 🞆Tabuk 🞆Eastern Province 🞆Northern Borders 🞆Jawf 🞆Makkah 🞆'Asir 🞆AL Baha 🞆Al Madinah 🞆Ha’il 🞆Najran 🞆 Jizan. 🞆 Outside KSA


 Health care sector: 🞆Ministry of Health H 🞆University H.🞆 Military H 🞆National Guard H 🞆King Faisal Specialist H. 🞆 Security Forces H. 🞆 Private H. 🞆 Other

Cath Lab Hospital: 🞆 yes 🞆 No
 🞆 STEMI 🞆 NSTEMI

Type of STEMI: 🞆 Anterior
 🞆 Inferior
 🞆 Other

Date of Admission : / /20

|  |  |  |
| --- | --- | --- |

Patient's Initials:

***** Patient's Phones : /

Date of birth: / /

Age (years): ! (Date of birth might not be accurate in some patients please make sure that you take the
 real age by asking the patient him or herself.)
 Gender: 🞆 Male 🞆 Female

Country of the hospital: choose one (Saudi Arabia, Qatar, Algeria, Bahrain, Egypt, Iraq, Jordan, Kuwait, Lebanon, Morocco, Oman, Tunisia, United Arab Emirates, Yemen, and Sudan)

*If country other than Saudi Arabia:

 Nationality of the Pt. : choose one (Qatar, Algeria, Bahrain, Egypt, Iraq, Jordan, Kuwait, Lebanon, Libya, Morocco, Oman, Tunisia, United Arab Emirates, Yemen, Sudan, other).

*****National ID

*****Or Passport No

Ethnicity: 🞆Arab 🞆 Other
 🞆 South Asian (India, Pakistan, Nepal, and Bangladesh)


 *If Saudi Arabia:

Nationality: 🞆Saudi 🞆No-Saudi

| 1 |  |  |  |  |  |  |  |  |  |
| --- | --- | --- | --- | --- | --- | --- | --- | --- | --- |

1- If Saudi
 ***** National ID:

2- If Non-Saudi:

| 2 |  |  |  |  |  |  |  |  |  |
| --- | --- | --- | --- | --- | --- | --- | --- | --- | --- |

*****If you have Iqama: Iqama No

*****If you do not have Iqama: passport No

Ethnicity: 🞆Arab 🞆 Other
 🞆 South Asian (India, Pakistan, Nepal, Bangladesh)

Height: cm

Weight: Kg

BMI:

**CAD Risk factors/Medical history:**

DM: 🞆 yes 🞆 No

HTN: 🞆 yes 🞆 No

Hypercholesterolemia: 🞆 yes 🞆 No

Current or ex-smoking: 🞆 yes 🞆 No

History of angina: 🞆 yes 🞆 No

History of MI: 🞆 yes 🞆 No

History of heart failure: 🞆 yes 🞆 No

History of PCI: 🞆 yes 🞆 No

History of CABG: 🞆 yes 🞆 No

History of stroke: 🞆 yes 🞆 No

History of chronic renal failure: 🞆 yes 🞆 No

**Part-2 pre-hospital care Section**

1- Chief complaint: a- (choose one)
 🞏 Chest pain
 🞏SOB/Fatigue
 🞏Epigastric/shoulder/back/neck pain 
 🞏 Cardiac arrest
 🞏other

b- Date / /20 time :

2-Transferred to your Emergency Department by an ambulance: 🞏Yes 🞏No

If yes,

Transferred by EMS e.g. Red Crescent or Red Cross? 🞏Yes 🞏No

! (**If the PT.is transferred to your hospital by EMS e.g. Red Crescent ambulance that is considered a medical contact before presenting to your hospital)**

3- Medical Contact before presenting to your hospital ⭘ yes ⭘ No

If yes,

A-

1**-** 1^st^ medical contact (choose one)

⭘EMS ⭘ an Emergency Department ⭘ Pharmacy ⭘ call a doctor / Clinic

-Date / / 20 time :

-choose one ⭘ Public ⭘ private

*****2- 2^nd^ medical contact (choose one)

⭘EMS ⭘ an Emergency Department ⭘ Pharmacy ⭘ call a doctor / Clinic

-choose one ⭘ Public ⭘ private

*****3- 3^rd^ medical contact (choose one)

⭘EMS ⭘ an Emergency Department ⭘ Pharmacy ⭘ call a doctor / Clinic

 -choose one ⭘ Public ⭘ private

B-Management at scene/ambulance/hospital:

CPR: ⭘ yes ⭘ No ⭘ Not available 

Intubation: ⭘ yes ⭘ No ⭘ Not available 

Aspirin ⭘ yes ⭘ No ⭘ Not available 

Nitro glycerin ⭘ yes ⭘ No ⭘ Not available 

ECG done ⭘ yes ⭘ No ⭘ Not available

If yes:

Date / / 20 time :

Transmitted to hospital (Fax, email, etc.): ⭘ yes ⭘ No

Thrombolytic given (outside of your hospital): ⭘ yes ⭘ No

If yes: Date / / 20 time :

**Part 3: Socioeconomic Status.**

1**-Education**
what level of formal education have you completed? Check the **highest** level

- None
- Primary
- Secondary/high school/Diploma(2years after high school)
- Trade school/vocational school.معاهد التدريب الفني والمدارس المهنية
- College/university
- Post-graduation degree. e.g. PHD, Master, Diploma

2- **Household income**

*****a- What is the average total monthly household income including subsidies? U.S.Dollars. (total income from each
 family member who share food, bills, insurance salary, disability income, retirement salary)

⭘ < 200$/month ⭘ 200-500$. ⭘ 500-2000$. ⭘ 2000-4000$ ⭘ >4000$

b- Total number of family members that you are responsible for financially?

c- Do you have difficulties with paying bills or buying food or clothes? 🞏Yes 🞏No,

If yes, how often did it happen in the last year? 🞏 All time 🞏 often 🞏 sometimes 🞏 rarely .

3-**Medical care.**

Are you covered by a private company medical insurance? 🞏Yes 🞏No

If no, are you covered by free governmental medical care? E.g. military hospitals. 🞏Yes 🞏No

If no, do you have difficulties to afford your medical care expenses? E.g. medications, operations, investigations.
 🞏Yes 🞏No

If yes, how often? 🞏 All time 🞏 often 🞏 sometimes 🞏 rarely .

4-**Occupation.**

Please indicate which group **best** describes your **current** occupation?

- Self-employed (as Independent, or have own business….)
- Employee (as salesperson, director, accountant…)
- Retired
- Unemployed (as housewife, househusband)

**Part 4: hospital section**

Emergency Department arrival: date / / 20 time : write the real arrival time not the time of registering the case in the file system

Status upon arrival

 HR: bpm
 SBP: mmgH
 DBP mmgH

Cardiac arrest: ⭘ yes 🞆 No

PT is admitted to: (choose one)
 ⭘ CCU/ICU ⭘ monitored ward bed ⭘ Unmonitored ward bed

 CHF Killip Class:
 1 (no CHF) 
 2 (rales and/or Jugular venous distension)
 3 (pulmonary edema)
 4 (cardiogenic shock)

ECG time: date / /20 time:

 Blood Investigations:

 *****- Peak Creatinine: µmol/L

 *****-Peak serum CK: IU/L

-Type of troponin available    🞆   cTn          🞆  hsTn

If (hsTn):
 1-type of (hsTn)     🞆   hsTnT     ! 🞆   hsTnI !

 2-Value …………pg/mL=ng/
 3- date and time / / 201 (only if NSTEMI)

Troponin: 🞆 +ve 🞆 -ve (+ve means the value of the Tn. Exceeds the AMI cut-off value of the Tn. Kits used)

If –ve plz, specify

*****-Choose one ⭘ BNP: ⭘ NT-pro BNP (optional)

Value pg/mL=ng/L or pmol/L

- Lowest Hb: g/dL

Cardiac examination tests:

1. Exercise stress test. 🞆performed 🞆not performed
   if performed:

Evidence of ischemia 🞆 yes 🞆 No

1. Stress Nuclear Scan. 🞆performed 🞆not performed

If performed:

Evidence of ischemia 🞆 yes 🞆 No

1. Dobutamine stress echo. 🞆performed 🞆not performed

If performed:

Evidence of ischemia 🞆 yes 🞆 No

1. CT-angiography. 🞆performed 🞆not performed

If performed:

Evidence of significant (>70% stenosis) coronary artery disease 🞆 yes 🞆 No.

Coronary angiography done**:** 🞆 Yes 🞆No

If yes: (Choose 1 option only)

1. Normal
2. Non-significant coronary artery disease
3. Significant Single-vessel disease
4. Significant Double-vessel disease
5. Significant Triple-vessel disease

*** Significant disease defined as Left Main stem lesion* ***more than 50%*** *and/or LAD or LCX or RCA or a branch more than 2.5mm in diameter has* ***more than 70% lesion or FFR less than 0.8****.*

Acute revascularization therapy:

A- If STEMI chosen from 1st page

Was the Pt. given a thrombolytic outside your hospital? 🞆Yes 🞆No

1- If yes,

Did the Pt. present to your hospital with clinical signs of reperfusion?       🞆Yes     🞆No

If No.

    Was thrombolytic therapy given inside your hospital?              🞆Yes     🞆No

     .a- If yes,

1-Thrombolytic therapy given Time: date: / /20 time:

2- Clinical Signs of Reperfusion 🞆 yes 🞆 No
 a- if yes,

Reperfusion achievement date: / /20 time:

Elective Cath done (**within 3-24 hrs. from reperfusion**) 🞆yes 🞆No

b- If No,

Rescue Cath +/- PCI 🞆yes 🞆No

If yes,

1-Arterial access: 🞆 Femoral 🞆 Radial 🞆 Brachial

2-Thrombectomy device used: 🞆 yes 🞆 No
 3- Choose all applied.

1. PCI to Left Main stem. 🞆yes 🞆No
2. PCI to LAD 🞆yes 🞆No
3. PCI to LCX 🞆yes 🞆No
4. PCI to RCA 🞆yes 🞆No
5. PCI to grafts/branches 🞆yes 🞆No

If No, Rescue Cath +/- PCI mention the reason……………………………

.b- If No,

Rescue Cath +/- PCI? 🞆 Yes 🞆 No

1- If yes:

1-Arterial access: 🞆 Femoral 🞆 Radial 🞆 Brachial

2-Thrombectomy device used: 🞆 yes 🞆 No
 3- Choose all applied

1. PCI to Left Main stem. 🞆yes 🞆No
2. PCI to LAD 🞆yes 🞆No
3. PCI to LCX 🞆yes 🞆No
4. PCI to RCA 🞆yes 🞆No
5. PCI to grafts/branches 🞆yes 🞆No

                       2- If No,

                   ((No thrombolytic therapy inside your hospital or Rescue Cath: why not? Pls, specify

2- If No,

Was Thrombolytic therapy given inside your hospital? 🞆 Yes 🞆 No

A. If yes:

1-Thrombolytic therapy given Time: date: / /20 time:

2- Clinical Signs of Reperfusion 🞆 yes 🞆 No
 .a- if yes,

Reperfusion achievement date: / /20 time:

Elective Cath done (**within 3-24 hrs. from reperfusion**) 🞆yes 🞆No

.b- If No,

Rescue Cath +/- PCI 🞆 yes 🞆 No

If yes:

1-Arterial access: 🞆 Femoral 🞆 Radial 🞆 Brachial

2-Thrombectomy device used: 🞆 yes 🞆 No
 3- Choose all applied.

1. PCI to Left Main stem. 🞆yes 🞆No
2. PCI to LAD 🞆yes 🞆No
3. PCI to LCX 🞆yes 🞆No
4. PCI to RCA 🞆yes 🞆No
5. PCI to grafts/branches 🞆yes 🞆No

If No, Rescue Cath +/- PCI mention the reason……………………………..

B. If No:

Primary PCI done: 🞆 yes 🞆 No ! (Ppci. means from Symptoms onset (chief complaint time) to first balloon inflation /device time ≤ 26 hrs.)

1-If yes:
 1- First balloon inflation/Device time: Date / / 20 time :

2-Arterial access: 🞆 Femoral 🞆 Radial 🞆 Brachial

3-Thrombectomy device used: 🞆 yes 🞆 No
 4- choose all applied.

1. PCI to Left Main stem. 🞆yes 🞆No
2. PCI to LAD 🞆yes 🞆No
3. PCI to LCX 🞆yes 🞆No
4. PCI to RCA 🞆yes 🞆No
5. PCI to grafts/branches 🞆yes 🞆No

2- If No:

No thrombolytic therapy or Primary PCI: Why not? Choose one

🞆Late presentation (≥24 hrs. from symptoms onset)
 🞆Contraindication (for thrombolytic)
 🞆 Missed
 🞆 Other

If other pls, specify

B- If NSTEMI chosen from 1st page.

Urgent/Emergency Cath: ⭘ yes ⭘ No

If yes: Arterial access: 🞆 Femoral 🞆 Radial 🞆 Brachial

Urgent/Emergency PCI done: ⭘ yes ⭘ No
 if yes,
 (Choose all applied)

1. PCI to Left Main stem. 🞆yes 🞆No
2. PCI to LAD 🞆yes 🞆No
3. PCI to LCX 🞆yes 🞆No
4. PCI to RCA 🞆yes 🞆No

E- PCI to grafts/branches 🞆yes 🞆No

Medications:

**First 24 hours of hospital Admission**

Aspirin: ⭘ yes ⭘ No

Clopidogrel: ⭘ yes ⭘ No

Prasugrel: ⭘ yes ⭘ No

Ticagrelor: ⭘ yes ⭘ No

Beta-Blockers: ⭘ yes ⭘ No

ACE-I or ARB: ⭘ yes ⭘ No

Statins: ⭘ yes ⭘ No

Aldosterone Inhibitor (Spironolactone): ⭘ yes ⭘ No

Heparins (UH or LMWH): ⭘ yes ⭘ No

GP 2b/3a inhibitors: ⭘ yes ⭘ No

Bivaluridin: ⭘ yes ⭘ No

Insulin: ⭘ yes ⭘ No

Oral hypoglycemic agents: ⭘ yes ⭘ No

Echo: 🞏 Yes 🞏 No

**If yes:**

🞏 Normal LV systolic function (EF >50%)

🞏 Mild LV systolic dysfunction (EF 40-50%)

🞏 Moderate LV systolic dysfunction (EF 30-40%)

🞏 Severe LV systolic dysfunction (EF <30%)

Major in-hospital Outcomes/Procedures:

| Recurrent ischemia | 🞏Yes 🞏No |
| --- | --- |
| Recurrent MI | 🞏Yes 🞏No |
| Atrial Fibrillation/Flutter: | 🞏Yes 🞏No |
| Heart Failure: | 🞏Yes 🞏No |
| Cardiogenic Shock: | 🞏Yes 🞏No |
| VT/VF arrest: | 🞏Yes 🞏No |
| IABP: | 🞏Yes 🞏No |
| Stroke: | 🞏Yes 🞏No |
| Major bleeding: | 🞏Yes 🞏No |
| Cardiac Tamponade: | 🞏Yes 🞏No |
| Stent thrombosis: | 🞏Yes 🞏No |
| elective coronary angiogram | 🞏Yes 🞏No |
| If yes, date. / /20 time: : |  |
| 1- Arterial access | 🞏 Femoral 🞏 Radial  🞏 Brachial |
| 2- Elective PCI: | 🞏Yes 🞏No |
| If yes : 1-Thrombectomy device used  2-(Choose all applied)   A- PCI to Left Main stem.  B- PCI to LAD  C- PCI to LCX  D- PCI to RCA  E- PCI to grafts/branches | 🞏Yes 🞏No  🞏Yes 🞏No  🞏Yes 🞏No  🞏Yes 🞏No  🞏Yes 🞏No  🞏Yes 🞏No |
| CABG  If yes: CABG option | 🞏Yes 🞏No  🞏 elective 🞏 emergency |

Discharge Status: 🞏 Alive 🞏 Dead

1- If alive:

Was the patient transferred to another hospital? ⭘ Yes ⭘ No

Medication at hospital discharge:

| Aspirin: | 🞏Yes 🞏No |
| --- | --- |
| Clopidogrel: | 🞏Yes 🞏No |
| Prasugrel: | 🞏Yes 🞏No |
| Ticagrelor: | 🞏Yes 🞏No |
| Beta-Blockers: | 🞏Yes 🞏No |
| ACE-I or ARB: | 🞏Yes 🞏No |
| Statins: | 🞏Yes 🞏No |
| Aldosterone Inhibitor  (Spironolactone) | 🞏Yes 🞏No |
| Oral Anticoagulants (Warfarin, Dabigatran,etc): | 🞏Yes 🞏No |
| Insulin: | 🞏Yes 🞏No |
| Oral hypoglycemic agents: | 🞏Yes 🞏No |

Date of Discharge: / / 20

2- If dead:

Date of death : / / 20

Online Final submission date: / /20 Time : (Generated by the system.)

CRF filled by …

Comments:

**AMI, CRF of non-Cath hospitals
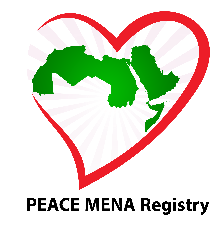
**

Part-1 Demographics

INCLUDE:

Consecutive patients hospitalized with Type 1 AMI (STEMI and NSTEMI) defined according to the ESC Guidelines, Age ≥ 18 years of age, written informed consent

EXCLUDE:

AMIs due to imbalance of oxygen supply and demand, AMIs resulting in deaths but without availability of biomarkers, and AMIs related to PCI or CABG (i.e.: AMI Types 2, 3, 4, and 5; respectively

*Denotes optional items.

PT. registry ID ! Automatically generated by the system when you fill the data from
 health care sector to ethnicity and click save. Write it back after you
 enroll the case.

*MRN:

Geographical. Region: 🞆Riyadh 🞆Qassim 🞆Tabuk 🞆Eastern Province 🞆Northern Borders 🞆Jawf 🞆Makkah 🞆'Asir 🞆AL Baha 🞆Al Madinah 🞆Ha’il 🞆Najran 🞆 Jizan. 🞆 Outside KSA

 Health care sector: 🞆Ministry of Health H 🞆University H.🞆 Military H 🞆National Guard H 🞆King Faisal Specialist H. 🞆 Security Forces H. 🞆 Private H. 🞆 Other

Cath Lab Hospital: 🞆 yes 🞆 No
 🞆 STEMI 🞆 NSTEMI

Type of STEMI: 🞆 Anterior
 🞆 Inferior
 🞆 Other

 Date of Admission: / /20

|  |  |  |
| --- | --- | --- |

Patient's Initials:

Date of birth: / /20


 Age (years): ¡ (date of birth might not be accurate in some patients please make sure that
 you take the real age by asking the patient him or herself).

 Gender: 🞆 Male 🞆 Female

Country of the hospital : choose one (Saudi Arabia, Qatar, Algeria, Bahrain, Egypt, Iraq, Jordan, Kuwait, Lebanon, Oman, Morocco, Tunisia, United Arab Emirates, Yemen, and Sudan).


 *If country other than Saudi Arabia:

 Nationality of the Pt. : choose one (Qatar, Algeria, Bahrain, Egypt, Iraq, Jordan, Kuwait, Lebanon, Libya, Oman, Morocco, Tunisia, United Arab Emirates, Yemen, Sudan, other).

*National ID
 *or Passport No

Ethnicity: 🞆Arab 🞆 Other
 🞆 South Asian (India, Pakistan, Nepal, and Bangladesh)


 *If Saudi Arabia:

Nationality: 🞆Saudi 🞆Non-Saudi

1- If Saudi:

| 1 |  |  |  |  |  |  |  |  |  |
| --- | --- | --- | --- | --- | --- | --- | --- | --- | --- |

*National ID:

2- If Non-Saudi:

| 2 |  |  |  |  |  |  |  |  |  |
| --- | --- | --- | --- | --- | --- | --- | --- | --- | --- |

*If you have Iqama: Iqama No
 .

*If you do not have Iqama: Passport No.

Ethnicity: 🞆Arab 🞆 Other
 🞆 South Asian (India, Pakistan, Nepal, and Bangladesh)

* Patient's Phones: /

Height: cm

Weight: Kg

BMI:

**CAD Risk factors/Medical history:**

DM: 🞆 yes 🞆 No

HTN: 🞆 yes 🞆 No

Hypercholesterolemia: 🞆 yes 🞆 No

Current or ex-smoking: 🞆 yes 🞆 No

History of angina: 🞆 yes 🞆 No

History of MI: 🞆 yes 🞆 No

History of heart failure: 🞆 yes 🞆 No

History of PCI: 🞆 yes 🞆 No

History of CABG: 🞆 yes 🞆 No

History of stroke: 🞆 yes 🞆 No

History of chronic renal failure: 🞆 yes 🞆 No

**Part-2 pre-hospital care Section**

1- Chief complaint: (choose one)
 🞏Chest pain
 🞏SOB/Fatigue
 🞏Epigastric/shoulder/back/neck pain 
 🞏Cardiac arrest
 🞏other

Date / /20 time :

**2-**Transferred to your Emergency Department by ambulance? 🞏Yes 🞏No

If yes,

Transferred by EMS e.g. Red Crescent or Red Cross? 🞏Yes 🞏No

(If the PT. Was transferred to your hospital by EMS e.g. Red Crescent ambulance that is considered a medical contact before presenting to your hospital)

**3-** Medical Contact before presenting to your hospital? ⭘ Yes ⭘ No

If yes,

A-

1**-** 1^st^ medical contact (choose one)

⭘EMS ⭘ an Emergency Department ⭘ Pharmacy ⭘ call a doctor / Clinic

-Date / / 20 time :

-choose one ⭘ Public ⭘ private

*2- 2^nd^ medical contact (choose one)

⭘EMS ⭘ an Emergency Department ⭘ Pharmacy ⭘ call a doctor / Clinic

-choose one ⭘ Public ⭘ private

*3- 3^rd^ medical contact (choose one)

⭘EMS ⭘ an Emergency Department ⭘ Pharmacy ⭘ call a doctor / Clinic

-choose one ⭘ Public ⭘ private

B-Management at scene/ambulance/hospital:

CPR: ⭘ yes ⭘ No ⭘ Not available 

Intubation: ⭘ yes ⭘ No ⭘ Not available 

Aspirin ⭘ yes ⭘ No ⭘ Not available 

Nitro glycerin ⭘ yes ⭘ No ⭘ Not available 

ECG done ⭘ yes ⭘ No ⭘ Not available 

If yes:

Date / / 20 time :

Transmitted to hospital (Fax, email, etc.): ⭘ yes ⭘ No

Thrombolytic given (outside of your hospital): ⭘ yes ⭘ No

If yes: Date / / 20 time :

**Part 3: Socioeconomic Status.**

1**-Education**
 what level of formal education have you completed? Check the **highest** level

- None
- Primary
- Secondary/high school/Diploma(2years after high school)
- Trade school/vocational school.معاهد التدريب الفني والمدارس المهنية
- College/university
- Post-graduation degree. e.g. PHD, Master, Diploma

2- **Household income**

*****a- What is the average total monthly household income including subsidies? U.S.Dollars. (total income from each family member who share food, bills, insurance salary, disability income, retirement salary)

⭘ < 200$/month ⭘ 200-500$. ⭘ 500-2000$. ⭘ 2000-4000$ ⭘ >4000$

b- Total number of family members that you are responsible for financially?

c- Do you have difficulties with paying bills or buying food or clothes? 🞏Yes 🞏No,

If yes, how often did it happen in the last year? 🞏 All time 🞏 often 🞏 sometimes 🞏 rarely .

3-**Medical care.**

Are you covered by a private company medical insurance? 🞏Yes 🞏No

If no, are you covered by free governmental medical care? E.g. military hospitals. 🞏Yes 🞏No

If no, do you have difficulties to afford your medical care expenses? E.g. medications, operations, investigations. 🞏Yes 🞏No

If yes, how often? 🞏 All time 🞏 often 🞏 sometimes 🞏 rarely

4-**Occupation.**

Please indicate which group **best** describes your **current** occupation?

- Self-employed (as Independent, or have own business….)
- Employee (as salesperson, director, accountant…)
- Retired
- Unemployed (as housewife, househusband)

**Part 4: hospital section**

Emergency Department arrival: date / / 20 time :

Status upon arrival

 HR: bpm

 SBP: mmgH

 DBP mmgH

Cardiac arrest: ⭘ yes 🞆 No

PT is admitted to: (choose one)
 ⭘ CCU/ICU ⭘ monitored ward bed ⭘ Unmonitored ward bed

 CHF Killip Class:
 1 (no CHF) 
 2 (rales and/or Jugular venous distension)
 3 (pulmonary edema)
 4 (cardiogenic shock)

ECG time: date / /20 time:

**Blood Investigations:**

 *- Peak Creatinine: µmol/L

 *-Peak serum CK: IU/L

 -Type of troponin available    🞆   cTn          🞆  hsTn

If (hsTn):
 1-type of (hsTn)     🞆   hsTnT     ! 🞆   hsTnI !

 2-Value …………pg/mL=ng/L
 3- date and time / / 201 : (only if NSTEMI)

Troponin: 🞆 +ve 🞆 -ve (+ve means the value of the Tn. Exceeds the AMI cut-off value of the Tn. Kits used)

If –ve plz, specify

*-Choose one ⭘ BNP: ⭘ NT-pro BNP (optional)

Value pg/mL=ng/L or pmol/L

 - Lowest Hb: g/d

Cardiac examination tests:

1. Exercise stress test. 🞆performed 🞆not performed
   if performed:

Evidence of ischemia 🞆 yes 🞆 No

1. Stress Nuclear Scan. 🞆performed 🞆not performed

If performed:

Evidence of ischemia 🞆 yes 🞆 No

1. Dobutamine stress echo. 🞆performed 🞆not performed

If performed:

Evidence of ischemia 🞆 yes 🞆 No

1. CT-angiography. 🞆performed 🞆not performed

If performed:

Evidence of significant (>70% stenosis) coronary artery disease 🞆 yes 🞆 No

**Acute revascularization therapy:**

A- If STEMI chosen from 1^st^ page.

Was the Pt. given a thrombolytic outside your hospital? ⭘Yes ⭘ No

1- If yes,

Did the Pt. present to your hospital with clinical signs of reperfusion?   ⭘Yes    ⭘ No

If No.

    Was thrombolytic therapy given inside your hospital?             ⭘ Yes    ⭘ No

      .a- If yes,

1-Thrombolytic therapy given date: / / 20 time:

2- Directly transferred to Cath-lab hospital (Drip & ship pathway)?
 ⭘ yes ⭘ No

.a- If No,

1-Mention the reason if No (Drip & ship pathway)……………………………………..

2-Clinical Signs of Reperfusion ⭘yes ⭘No

a- If yes,

. Reperfusion achievement date: / / 20 time:

Transferred for elective Cath (**within 3-24 hrs. from reperfusion**)
 ⭘ yes ⭘ No

.b- If No. Transferred for Rescue Cath +/- PCI? ⭘ yes ⭘ No

If No (Transferred for Rescue Cath +/- PCI), mention the reason

.b- If yes,

             1- (Choose one)

                       ⭘1-Pt transferred ≤90 mint from thrombolytic given time

                       ⭘2- Pt transferred >90 mint from thrombolytic given time

             2- Reperfusion achieved before transfer   ⭘ yes              ⭘No

.b- If No,

Transferred for rescue Cath+/- PCI?        ⭘Yes        ⭘No

              If No, (No thrombolytic therapy inside your hospital or transferred for Rescue Cath: why not? Pls, specify:

2- If No,

Was thrombolytic therapy given inside your hospital?              ⭘Yes     ⭘No

  .a- If yes,

1-Thrombolytic therapy given date: / / 20 time: :

2-Directly transferred to Cath-lab hospital (Drip & ship pathway) ⭘yes ⭘No

.a-If No,

1-Mention the reason of No (Drip & ship pathway)………………………………..

2-Clinical Signs of Reperfusion ⭘yes ⭘No

.a- If No.

Transferred for Rescue Cath +/- PCI? ⭘ Yes ⭘ No

If No (Transferred for Rescue Cath +/- PCI?) mention the
 reason……………

.b- If yes,

Reperfusion achievement date: / / 20 time:

Transferred for elective Cath (within 3-24 hrs. from reperfusion)
 ⭘yes ⭘No

.b-If yes,

             a- (Choose one)

                       ⭘1-Pt transferred ≤90 mint from thrombolytic given time

                       ⭘ 2- Pt transferred >90 mint from thrombolytic given time

             b- Reperfusion achieved before transfer  ⭘yes     ⭘No.

b- If No,

Transferred for primary PCI? ⭘ Yes ⭘No ! (Symptoms onset to ER arrival ≤ 24 hrs.)

If No, No thrombolytic therapy or Transferred for Primary PCI: Why not? (Choose one)
 🞆 Late presentation (≥24 hrs. from symptoms onset)
 🞆Contraindication (for thrombolytic)
 🞆 Missed
 🞆 Other If other Pls, Specify

B- If NSTEMI chosen from 1st page.

Transferred to another hospital for Urgent coronary angiogram: ⭘ yes ⭘ No

**Medications:**

**First 24 hours of hospital Admission :**

Aspirin: ⭘ yes ⭘ No

Clopidogrel: ⭘ yes ⭘ No

Prasugrel: ⭘ yes ⭘ No

Ticagrelor: ⭘ yes ⭘ No

Beta-Blockers: ⭘ yes ⭘ No

ACE-I or ARB: ⭘ yes ⭘ No

Statins: ⭘ yes ⭘ No

Aldosterone Inhibitor (Spironolactone): ⭘ yes ⭘ No

Heparins (UH or LMWH): ⭘ yes ⭘ No

GP 2b/3a inhibitors: ⭘ yes ⭘ No

Bivaluridin: ⭘ yes ⭘ No

Insulin: ⭘ yes ⭘ No

Oral hypoglycemic agents: ⭘ yes ⭘ No

**Echo: 🞏 Yes 🞏 No**

**If yes:**

🞏 Normal LV systolic function (EF >50%)

🞏 Mild LV systolic dysfunction (EF 40-50%)

🞏 Moderate LV systolic dysfunction (EF 30-40%)

🞏 Severe LV systolic dysfunction (EF <30%)

**Major in-hospital Outcomes/Procedures:**

| Recurrent ischemia | 🞏Yes 🞏No |
| --- | --- |
| Recurrent MI | 🞏Yes 🞏No |
| Atrial Fibrillation/Flutter: | 🞏Yes 🞏No |
| Heart Failure: | 🞏Yes 🞏No |
| Cardiogenic Shock: | 🞏Yes 🞏No |
| VT/VF arrest: | 🞏Yes 🞏No |
| IABP: | 🞏Yes 🞏No |
| Stroke: | 🞏Yes 🞏No |
| Major bleeding: | 🞏Yes 🞏No |
| Cardiac Tamponade: | 🞏Yes 🞏No |
| stent thrombosis | 🞏Yes 🞏No |
| Transferred for elective coronary angiogram. | 🞏Yes 🞏No |
| If yes, | Date: / /20 time: : |

Discharge Status: 🞏 Alive 🞏 Dead
**1-If alive:**

Was the patient transferred to another hospital? ⭘ Yes ⭘ No

Medication at hospital discharge:

| Aspirin: | 🞏Yes 🞏No |
| --- | --- |
| Clopidogrel: | 🞏Yes 🞏No |
| Prasugrel: | 🞏Yes 🞏No |
| Ticagrelor: | 🞏Yes 🞏No |
| Beta-Blockers: | 🞏Yes 🞏No |
| ACE-I or ARB: | 🞏Yes 🞏No |
| Statins: | 🞏Yes 🞏No |
| Aldosterone Inhibitor (Spironolactone) | 🞏Yes 🞏No |
| Oral Anticoagulants (Warfarin, Dabigatran,etc): | 🞏Yes 🞏No |
| Insulin: | 🞏Yes 🞏No |
| Oral hypoglycemic agents: | 🞏Yes 🞏No |

Date of Discharge**: / / 20**

**2-If dead:**

Date of death : / / 20

Online Final submission date: / /20 time : ! (Generated by the system).

CRF filled by …

Comments:

**Follow-Up section**

1- (1-Month follow up)

1-month from date of discharge.

Hospital re-admission and mortality follow up-

The PT is 🞆reachable 🞆unreachable (lost)

1- If unreachable, Date of last call: / /20 dd/mm/yyyy

2- If reachable,

Date of call/clinic visit for 1- month: / /20 dd/mm/yyyy

1-month-re-admission 🞆No 🞆 yes 🞆 unknown

If yes, date of the last re-admission: / /20 dd/mm/yyyy

Cause: 🞆Cardiac cause 🞆 Non-cardiac cause 🞆unknown

1-Month mortality 🞆Alive 🞆 Dead

If dead date of death: / /20 dd/mm/yyyy

Cause: 🞆Cardiac cause 🞆 Non-cardiac cause 🞆unknown

Online 1-month final submission date/time: / /20 time. :
 given by the system when click final subit.

(1-year follow up)

1-year from date of discharge

A- Hospital re-admission and mortality follow up

The PT is 🞆reachable 🞆unreachable (lost)

1- If unreachable, Date of last call: / /20 dd/mm/yyyy

2- If reachable,

Date of call/clinic visit for 1- year: / / 20 dd/mm/yyyy

1-year re-admission 🞆No 🞆 yes 🞆 unknown

If yes date of the last re-admission: / /20 dd/mm/yyyy

Cause: 🞆Cardiac cause 🞆 Non-cardiac cause 🞆unknown

1-year mortality 🞆Alive 🞆 Dead

If dead date of death: / /20 dd/mm/yyyy

Cause: 🞆Cardiac cause 🞆 Non-cardiac cause 🞆unknown

B- Medications, PCI and CABAG**.**

Aspirin 🞆yes 🞆No 🞆unknown

 Clopidogrel 🞆yes 🞆No 🞆unknown

Ticagrelor  🞆yes 🞆No 🞆unknown

Statin  🞆yes 🞆No 🞆unknown

Beta-blocker 🞆yes 🞆No 🞆unknown

ACE-I or ARB 🞆yes 🞆No 🞆unknown

Mineralocorticoid receptor antagonist (Spironolactone) 🞆yes 🞆No 🞆unknown

Oral anticoagulant (warfarin or NOACs)       🞆yes 🞆No 🞆unknown

Current Smoking or Shisha: 🞆yes 🞆No 🞆unknown

Repeat Coronary angiogram: 🞆yes 🞆No 🞆unknown
 Try to get Cath-report if not possible ask the pt. if a stent was put.
 - If Yes: Date: mm/yyyy

PCI: 🞆yes 🞆No 🞆unknown
 if yes Date: dd/mm/yyyy if you do not know the exact day choose 1 for the day.

CABG: 🞆yes 🞆No 🞆unknown
 if yes Date: dd/mm/yyyy if you do not know the exact day choose 1 for the day

Hospital admission due to Recurrent Angina or Myocardial Infarction: 🞆yes 🞆No 🞆unknown
 If Yes: Date: dd/mm/yyyy if you do not know the exact day choose 1 for the day

*****BP: mmgH (within 2 weeks before or after date of call or clinic visit)

Systolic Diastolic e.g. 090

*****Fasting Blood Glucose: (Within 2 weeks before or after date of call or clinic visit)
Mmol/L: Mmol/L= 0.0555 X mg/dL

mg/dL:

*****Fasting LDL: (within 2 weeks before or after date of call)
Mmol/L: Mmol/L= 0.0259 X mg/dL

mg/dL:

Online 1-year final submission date/time: / /20 time. :
 given by the system when click final submit.


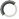

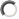

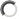

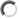

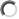

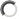

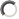

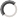

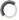

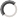

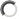

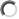

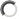

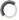

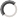

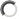

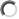

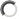

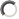

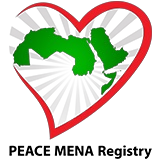


Acute Heart Failure CRF.

Demographic/Admission Information

- .Patient Initials:
- .Registry ID:

- .Patient Telephone No.1 (Without country/area code) :
- .Patient Telephone No.2 (Without country/area code) :

(Optional)

- .Next of Kin Telephone No. :
- .Age: Years
- .Gender Male Female
- .Nationality MENA Others ALGERIA BAHRAIN

EGYPT IRAQ JORDAN KUWAIT LEBANON MOROCCO OMAN

Palestine Syria QATAR

SAUDI ARABIA SUDAN TUNISIA

UAE YEMEN

.

Admission Date:

- .Emergency admission time :

  hospital type:
   🞆cath or🞆 Non-Cath

D D M M Y Y Y Y

: 24 Hrs

Socioeconomic section:

1. - Education

What level of formal education have you completed? Check the highest level

None

Primary

Secondary/high school/Diploma (2years after high school)

Trade school/vocational school. معاهد التدريب الفني والمدارس المهنية

College/university

Post-graduation degree. E.g. PHD, Master, Diploma

1. - Household Income

- - What is the average total monthly household income including subsidies? U.S.Dollars. (total income from each family member who share food, bills, insurance salary, disability income, retirement salary) (OPTIONAL)

< 200 $ monthly

200-500 $ monthly

500-2000 $ monthly

2000-4000 $ monthly

>4000 $ monthly

- - Total number of family members that you are responsible for, financiallly?
- -Did you find difficulties with paying bills or buying food or clothes in the last year?

If yes:

Yes No

All Time

Often

Sometimes

Rarely

1. Medical Care

- -Are you covered by a private company medical insurance? Yes No If yes go to Question 4

if No i Are you covered by free governmental medical care? (e.g., military hospitals)


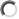

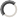

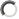

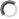

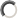

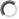

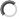

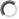

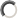

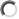

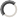

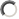

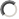

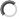

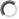

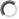

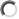

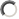

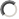

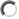

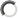


Yes No If yes go to Question 4

if No ii do you have difficulties to afford your medical care expenses? (e.g., meications, operatios, investigations)

Yes No (If No go to Question 4)


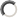

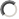

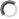

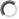

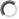

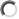

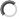

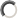

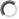

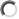

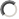

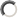

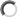

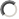

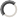

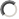

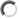

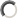

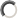


If yes how often did it happen in the last year?

All Time

Often

Sometimes

Rarely

1. Occupation

- -Please indicate which group best describes your current occupation?

Self employed (as Independent, or have own business....)

Employee (as salesperson, director, accountant…)

Retired

Unemployed (as housewife, househusband)

- .Brought by: Ambulance Others (Self, Relative or Friend)
- .Admitted to: CCU/ICU Monitored Bed Unmonitored Bed
- .Main Care Giver: Cardiologist Internist
- .Type of HF: Acute De novo HF( first time) Acute on chronic HF


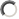

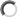

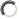

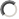

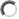

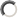

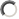

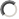

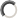

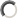
Medical History


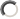

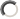

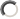

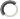

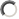

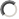

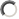

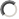

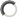

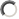

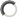

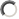

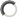

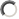

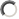

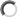


**Does Patient have a history or documentation of: Select under Yes or No**

1. Past Cardiovascular History:
   1. Heart Failure: Yes No
   2. IHD (angina or MI). Yes No

If yes :

- . Documented Coronary angiogram in the past : Yes No
- . PCI : Yes No
- . CABG : Yes No
- . Device therapy : Yes No
  - 1. CRT-P :
    2. CRT-D :
    3. ICD :
    4.
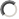

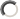

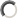

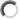

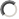

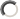

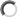

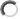

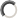

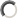
PPM :

| - . Valve Repair/ Replacement | : |  | Yes | No |
| --- | --- | --- | --- | --- |
| Mitral : Aortic : Tricuspid : Pulmonic : | Yes Yes Yes Yes | No No No No |  |  |
| 3. Valvular Heart Disease : |  |  | Yes | No |
| 4. Congenital Heart Disease : |  |  | Yes | No |
| 5. Atrial Fibrillation : |  |  | Yes | No |
| 6. PVD : |  |  | Yes | No |
| 7. Stroke/ TIA : |  |  | Yes | No |

1. Risk factors:


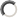

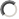


- 1. Current Smoking : Yes No

Choose one or more: Cigarettes. Yes No

Water pipe. Yes No

Cigar Pipe. Yes No Chewing Tobacco Yes No

- 1. Khatt : Yes No
  2. Alcohol : Daily Occasionally Never
  3. Known DM : Yes No

Type 1 DM

Type 2 DM

- 1. Known HTN : Yes No
  2. Known hyperlipidemia : Yes No
  3. Family history of Cardiomyopathy/ Heart failure : Yes No
  4. Peripartum : Yes No
  5. CKD / Dialysis : Yes No
  6. Sleep Apnea requiring therapy : Yes No
  7. Others : Yes No

Choose one or more:

Radiation. Yes No

Chemotherapy Yes No

Thyroid disorder Yes No

Asthma/ COPD Yes No

Clinical Presentation

Cardiac Arrest on Arrival: Yes No

Shock: Yes No

Choose one

Cardiogenic shock

Non cardiogenic shock (septic)

- . NYHA Class : NYHA I

NYHA II

NYHA III

NYHA IV

**B . Signs**

HR: Beats/min

BP(mmHg) : Systolic

Diastolic

RR : /min

1. Pulse Oxymetry Saturation % (optional)
   1. Weight : Kg
   2. Height : Cm
   3. BMI : . kg/m2

Investigations at Presentation

- . Troponin-I/T : Elevated Normal Not Done
- . Type of troponin available : cTn hsTn
- . Type of (hsTn) : hsTnT hsTnI
- .Value : pg/mL=ng/L
- .BNP :

Yes No

pg/ml=ng/L OR pmol/L

- .NT-Pro BNP :

Yes No

pg/ml=ng/L OR pmol/L

- . First Blood Glucose:

. mg/dl

. mmoI/L

- . HbA1c: . % (optional)
- . Total Cholesterol :

. mg/dl (optional)

. mmol/L

- . First Hemoglobin: . g/dL
- . First WBC: . x109/µL
- . First Urea :

. mg/dl

. mmol/L

- . First Creatinine :

. mg/dL

. umol/L

- . E-GFR : . ml/min
- . First Serum Sodium : . mmol/L or mEq/L
- . First Serum Potassium : . mmol/Lit or mEq/L
- . First ALT : units/l
- . Exercise stress test : Performed Not Performed
- . Evidence of ischemia: Yes No
- . Stress Nuclear Scan. :: Performed Not Performed
- . Evidence of ischemia: Yes No
- . Dobutamine stress echo : Performed Not Performed
- . Evidence of ischemia: Yes No
- . CT - angiography. : Performed Not Performed
- . Evidence of significant (>70% stenosis) coronary artery disease: Yes No
- . First ECG :
  - 1. Sinus AF/Flutter Others

vii . QRS Duration = > 0.12 Yes No

msec: **Choose one**

LBBB

RBBB

- . Echocardiography Information : Yes No

Normal LV function( EF>50%).

Mild LV dysfunction( EF:40-50%).

Moderate LV dysfunction( EF:30-39%).

Severe LV dysfunction( EF < 30%).

1. Moderate to Severe Valve Disease :

Yes No Choose one or more: MS: Yes No

MR: Yes No

AS: Yes No

AR: Yes No

TR: Yes No

- . PA systolic pressure: . mmHg (optional)

Medications

- . IV Frusemide boluses : Yes No
- . IV Frusemide Infusion : Yes No
- . IV first emergency Frusemide boluses/IV Frusemide Infusion Date:

D D M M Y Y Y Y

- . Time of first emergency IV Frusemide administration :

: 24 Hrs

- . IV Nitrates : Yes No

- . Inotropes : Yes No

Before Admission On Discharge

- - . Status on discharge: Alive Death
- . furosemide :

Yes No Yes No

- . Digoxin :

Yes No Yes No

- . Oral Nitrates :

Yes No Yes No

- . Calcium blockers :

Yes No Yes No

- . hydralazine :

Yes No Yes No

- . Antiplatelet :

Yes No Yes No

- . Oral Anticoagulants :

Yes No Yes No

- . If Oral Anticoagulants Before Admission (YES)Choose one:

Warfarin NOACS

- . If Oral Anticoagulants on discharge (YES) Chose one :

Warfarin NOACS

- . Heparin/LMWH :

Yes No Yes No

- . Statin :

Yes No Yes No

- . Allopurinol :

Yes No Yes No

- . Ivabradine :

Yes No Yes No

- . Antiarrhythmic drug :

Yes No Yes No

- . Antidepressants :

Yes No Yes No

- . BB :

Yes No Yes No

1. Carvedilol : 1. Carvedilol :

| Dose | Frequency | Dose | | Frequency |
| --- | --- | --- | --- | --- |
| 3.125 | OD | 3.125 | | OD |
| 6.25 | BID | 6.25 | | BID |
| 12.5 | TID | 12.5 | | TID |
| 25 | QID | 25 | | QID |
| 2. Bisoprolol : |  | 2. Bisoprolol : | |  |
| Dose | Frequency | Dose | | Frequency |
| 1.25 | OD | 1.25 | | OD |
| 2.5 | BID | 2.5 | | BID |
| 3.75 | TID | 3.75 | | TID |
| 5 | QID | 5 | | QID |
| 7.5 |  | 7.5 | |  |
| 10 |  | 10 | |  |
| 3. Metaprolol : |  | 3. Metaprolol : | |  |
| Dose | Frequency | Dose | | Frequency |
| 12.5 | OD | 12.5 | | OD |
| 25 | BID | 25 | | BID |
| 50 | TID | 50 | | TID |
| 100 | QID | 100 | | QID |
| 200 |  | 200 | |  |
| 4. Atenolol : |  | 4. Atenolol : | |  |
| Dose | Frequency | Dose | | Frequency |
| 12.5 | OD | 12.5 | | OD |
| 25 | BID | 25 | | BID |
| 50 | TID | 50 | | TID |
| 100 | QID | 100 | | QID |
| 200 |  | 200 | |  |
| S. ACE- Inhibitors : | | | | |
| Yes | No Yes | No |  |  |
| 1. Captopril : |  |  | 1. Captopril : |  |
| Dose | Frequency |  | Dose | Frequency |
| 3.12 | OD |  | 3.12 | OD |
| 6.25 | BID |  | 6.25 | BID |
| 12.5 | TID |  | 12.5 | TID |
| 25 | QID |  | 25 | QID |
| 50 |  |  | 50 |  |

Lisinopril : 2. Lisinopril :

Dose Frequency Dose Frequency

2.5

5

10

20

OD BID TID QID

2.5

5

10

20

OD BID TID QID

1. Enalapril : 3. Enalapril :

Dose Frequency Dose Frequency

2.5

5

10

20

OD BID TID QID

2.5

5

10

20

OD BID TID QID

1. Perindopril : 4. Perindopril :

Dose Frequency Dose Frequency

2 OD

1. BID
2. TID

8 QID

10

16

2 OD

1. BID
2. TID

8 QID

10

16

1. Ramipril : 5. Ramipril :

Dose Frequency Dose Frequency

1.25

2.5

5

10

OD BID TID QID

1.25

2.5

5

10

OD BID TID QID

1. Fosinopril : 6. Fosinopril :

Dose Frequency Dose Frequency

5 OD

10 BID

15 TID

20 QID

5 OD

10 BID

15 TID

20 QID

1. Cilazapril : 7. Cilazapril :

Dose Frequency Dose Frequency

0.5

1

2.5

5

OD BID TID QID

0.5

1

2.5

5

OD BID TID QID

1. ARBs :

Yes No Yes No

- 1. Valsartan : 1. Valsartan :

| Dose | Frequency | Dose | Frequency |
| --- | --- | --- | --- |
| 40 | OD | 40 | OD |
| 80 | BID | 80 | BID |
| 160 | TID | 160 | TID |
|  | QID |  | QID |

- 1. Candesartan : 2. Candesartan :

| Dose | Frequency | Dose | Frequency |
| --- | --- | --- | --- |
| 4 | OD | 4 | OD |
| 8 | BID | 8 | BID |
| 16 | TID | 16 | TID |
| 32 | QID | 32 | QID |

- 1. Losartan : 3. Losartan :

| Dose | Frequency | Dose | Frequency |
| --- | --- | --- | --- |
| 25 | OD | 25 | OD |
| 50 | BID | 50 | BID |
| 100 | TID | 100 | TID |
|  | QID |  | QID |

- 1. Telmesartan : 4. Telmesartan :

| Dose | Frequency | Dose | Frequency |
| --- | --- | --- | --- |
| 20 | OD | 20 | OD |
| 40 | BID | 40 | BID |
| 80 | TID | 80 | TID |
|  | QID |  | QID |

- 1. Irbesartan : 5. Irbesartan :

| Dose | Frequency | Dose | Frequency |
| --- | --- | --- | --- |
| 75 | OD | 75 | OD |
| 150 | BID | 150 | BID |
| 300 | TID | 300 | TID |
|  | QID |  | QID |

- . Aldosterone Antagonists

Yes No Yes No

1. Spironolactone : 1. Spironolactone :

| Dose | Frequency | Dose | Frequency |
| --- | --- | --- | --- |
| 12.5 | OD | 12.5 | OD |
| 25 | BID | 25 | BID |
| 50 | TID | 50 | TID |
| 100 | QID | 100 | QID |

1. Eplerenone : 2. Eplerenone :

| Dose | Frequency | Dose | Frequency |
| --- | --- | --- | --- |
| 25 | OD | 25 | OD |
| 50 | BID | 50 | BID |
|  | TID | http://192.168.0.105/CUS/peace/crfs/index | TID |
|  | QID |  | QID |

- . Entresto (sacubitril/valsartan) before admission
- . Entresto (sacubitril/valsartan) On Discharge:

Yes No Yes No 50mg

100mg 200mg

50mg

100mg 200mg

Cardiac Procedures

- . Coronary Angiogram: Yes No

Normal

Non-significant coronary artery disease

Significant single vessel disease

Significant double vessel disease

Significant triple vessel disease

** Significant disease defined as Left Main stem lesion more than 50% and/or LAD or LCX or RCA or a branch more than 2.5mm in diameter has more than 70% lesion or FFR less than 0.8.

- . PCI : Yes No
- . PCI to Left Main stem. :

Yes No

| - . PCI to LAD : - . PCI to LCX : - . PCI to RCA : | Yes  Yes Yes | No  No No |
| --- | --- | --- |
| - . PCI to grafts/branches : | Yes | No |
| - . CABG : | Yes | No |
| - . Device therapy : | Yes | No |
| i. CRT-P : |  |  |

- 1. CRT-D :
  2. ICD :
  3. PPM :
- .
- Valve Repair/ Replacement : Yes No

Mitral : Yes No

Aortic : Yes No

Tricuspid : Yes No

Pulmonic : Yes No

In-Hospital Course

| ●. | Intubation/ Ventilation : | Yes | No |
| --- | --- | --- | --- |
| ●. | IABP : | Yes | No |
| ●. | Acute Dialysis/ Ultrafiltration : | Yes | No |
| ●. | VT/VF Requiring Therapy : | Yes | No |
| ●. | AF Requiring Therapy : | Yes | No |
| ●. | Major bleeding : | Yes | No |
| ●. | Blood Transfusion : | Yes | No |
| ●. | Stroke : | Yes | No |
| ●. | Systemic Infection Requiring Antibiotics : | Yes | No |

**Discharge Information**

- . HR : Beats/min
- BP(mmHg) : Systolic Diastolic
- . Weight : Kg
- . NYHA Class : NYHA I

NYHA II

NYHA III

NYHA IV

- .What is the main cause of heart failure:

1. Choose one

ISCHEMIC HEART DISEASE

PRIMARY VALVE DISEASE

HYPERTENSIVE HEART DISEASE

HYPERTROPHIC CMP

MYOCARDITIS

IDIOPATHIC CMP

CARDIOTOXIC CMP

RIGHT SIDED HEART FAILURE

PREGNANCY RELATED CMP

OTHERS

1. The main precipitating factor that leading to this Acute Heart Failure: **(Choose one most probable cause)**
   1. Noncompliance with medications
   2. Noncompliance with Diet
   3. Salt Retaining Drugs (NSAIDs)
   4. Acute coronary syndrome
   5. Uncontrolled hypertension
   6. Uncontrolled arrhythmias
   7. Anemia
   8. Infection
   9. Worsening Renal Failure
   10. Pulmonary embolism
   11. Unknown

- . Discharged :

1. Home
2. discharged Against Medical Device
3. Transferred to another Hospital
4. Death

- . Date of discharge/transfer/death :

D D M M Y Y Y Y

- . Full name of Physician who filled the form :

Firstname: Middlename: Famliyname:

- . Date of Final submission date:

D D M M Y Y Y Y

30 days Followup

- . The Patient is Reachable Unreachable
- . If Unreachable Date Of Telephone call :

D D M M Y Y Y Y

If Reachable:

- . Date of call/clinic visit for 1 -month :

D D M M Y Y Y Y

. Alive Yes No

- . Re-hospitalization with AHF requiring intravenous diuretic treatment

Yes No

- . Stroke after discharge Yes No
- . Date of stroke after discharge :

D D M M Y Y Y Y

- . MI after discharge Yes No
- . If MI after discharge yes: date :

D D M M Y Y Y Y

- . Emergency revascularization for acute ischemia or MI :

Yes No

If Emergency revascularization for acute ischemia or MI: yes, date :

D D M M Y Y Y Y

- . if Died Approximate Date of Death :

D D M M Y Y Y Y

- . Full name of Physician who filled the form :

Firstname: Middlename: Famliyname:

12 Month Followup

- . The Patient is Reachable Unreachable
- . If Unreachable Date Of Telephone call :

D D M M Y Y Y Y

If Reachable:

- . Date of call/clinic visit for 12-month :

D D M M Y Y Y Y

- . Alive Yes No
- . Re-hospitalization with AHF requiring intravenous diuretic treatment

Yes No

- . ICD/ CRT-D Yes No
- . PCI/ CABG Yes No
- . Medications:

1. BB Yes No
2. ACE- Inhibitors Yes No
3. ARBs Yes No
4. Aldosterone Antagonists Yes No
5. Diuretics Yes No
6. Digoxin Yes No
7. Ivabradine Yes No
8. Statins Yes No
9. Entresto(sacubitril/valsartan) Yes No

- . If Died Approximate Date of Death :

D D M M Y Y Y Y
Full name of Physician who filled the form : Firstname: Middlename: Famliyname
