# Supplementary material for: Acute myocardial infarction and acute heart failure in the Middle East and North Africa: Study design and pilot phase study results from the PEACE MENA registry
Source: PLoS One. 2020 Jul 22;15(7):e0236292. doi: 10.1371/journal.pone.0236292 (PMC7375595; doi:10.1371/journal.pone.0236292)
Supplement: S1 Table — (DOCX) [file pone.0236292.s002.docx]

**S 1 Table.**
 **Educational level and socio-economic status in the ACS population**

| **Education** | **Total** |
| --- | --- |
| None | 73/468 (15.60%) |
| Primary | 97/468 (20.73%) |
| Secondary/high school/Diploma | 136/468 (29.06%) |
| Trade/vocational school | 25/468 (5.34%) |
| College/university | 120/468 (25.64%) |
| Post-graduation degree. e.g. PHD, Master | 17/468 (3.63%) |
| Average total monthly household income including subsidies | Please do the same as for AHF table |
| Monthly Income, Median (IQR) | 600.0 (700.0) |
| Monthly Income |  |
| < 500 $ | 161(38.4%) |
| 500-2000 $ | 196(46.8%) |
| 2000-4000 $ | 30(7.2%) |
| >4000 $ | 32(7.6%) |
| Family members (who live in your house), Median (IQR) | 5.00 (2.00) |
| **Difficulties in paying bills or buying food or clothes in the last year** |  |
| All Time | 15/125 (12.00%) |
| Often | 47/125 (37.60%) |
| Sometimes | 49/125 (39.20%) |
| Rarely | 12/125 (9.60%) |
| Never | 2/125 (1.60%) |
| **Coverage of medical care expenses** |  |
| Difficulties to afford medical care expenses | 102/177 (57.63%) |
| Coverage by a private medical insurance | 76/474 (16.03%) |
| Coverage by free governmental medical care | 221/398 (55.53%) |
| **occupation** |  |
| Self-employed (as Independent, or have own business) | 108/489 (22.09%) |
| Employee (as salesperson, director, accountant) | 189/489 (38.65%) |
| Retired | 100/489 (20.45%) |
| Unemployed (as housewife, househusband) | 92/489 (18.81%) |
