# Supplementary material for: Acute myocardial infarction and acute heart failure in the Middle East and North Africa: Study design and pilot phase study results from the PEACE MENA registry
Source: PLoS One. 2020 Jul 22;15(7):e0236292. doi: 10.1371/journal.pone.0236292 (PMC7375595; doi:10.1371/journal.pone.0236292)
Supplement: S2 Table — (DOCX) [file pone.0236292.s003.docx]

**S2 Table**

Clinical features, management, and outcomes of patients with lower versus higher income presenting with acute myocardial infarction

|  | lower income (<500$/month)  n= 198 (47.26 %) | Higher income (>500$/month)  n=221(52.74 %) | P-value |
| --- | --- | --- | --- |
| Age | 57.27 ± 12.50 | 57.12 ± 11.21 | 0.897 |
| Male | 162 (81.82%) | 201 (90.95%) | 0.006 |
| STEMI/NSTEMI |  |  | 0.077 |
| STEMI | 127 (64.14%) | 123 (55.66%) |  |
| NSTEMI | 71 (35.86%) | 98 (44.34%) |  |
| Residency |  |  | <.001 |
| GCC country (KSA, Kuwait, Qatar, Bahrain, UAE, Oman) | 21 (10.61%) | 65 (29.41%) |  |
| Non-GCC country (all other countries together) | 177 (89.39%) | 156 (70.59%) |  |
| Low education (none, primary, secondary) | 165 (83.33%) | 98 (44.34%) | <.001 |
| **Medical history** |  |  |  |
| HTN | 101 (51.01%) | 121 (54.75%) | 0.444 |
| Diabetes | 89 (44.95%) | 120 (54.30%) | 0.056 |
| Current or ex-smoker | 124 (62.63%) | 123 (55.66%) | 0.148 |
| Dyslipidemia | 65 (32.83%) | 103 (46.61%) | 0.004 |
| Prior angina or MI | 85 (42.93%) | 69 (31.22%) | 0.013 |
| Prior PCI | 24 (12.12%) | 40 (18.10%) | 0.089 |
| Prior CABG | 5 (2.53%) | 9 (4.07%) | 0.379 |
| Heart Failure | 11 (5.56%) | 9 (4.07%) | 0.477 |
| Stroke | 16 (8.08%) | 3 (1.36%) | <.001 |
| Chronic kidney disease | 8 (4.04%) | 8 (3.62%) | 0.823 |
| **Clinical presentation** |  |  |  |
| HR>100 bpm | 32 (16.16%) | 20 (9.05%) | 0.027 |
| BP< 90 mmHg | 5 (2.53%) | 5 (2.26%) | 0.860 |
| Killip class 3 or 4 HF | 15 (7.58%) | 10 (4.52%) | 0.188 |
| **Cardiac Procedures** |  |  |  |
| Echo: Moderate or severe LV dysfunction | 36 (20.69%) | 35 (19.02%) | 0.692 |
| Elective or emergency PCI | 55 (60.44%) | 75 (64.10%) | 0.588 |
| Primary PCI for STEMI  If yes: door-to-balloon time <90 minutes, n (%)  If yes: door-to-balloon time <90 minutes, n (%) | 54 (60.67%) | 79 (79.00%) | 0.006 |
|  | 33 (61.11%) | 57 (72.15%) | 0.181 |
| CABG | 5(2.53%) | 11(4.98%) | 0.212 |
| **Medications at discharge** |  |  |  |
| Anti-platelets (Aspirin, Clopidogrel, Ticagrelor) | 194 (100.00%) | 218 (99.54%) | 0.346 |
| Beta-blockers | 178 (91.75%) | 198 (90.41%) | 0.634 |
| ACE-Is/ARBs | 175 (90.21%) | 172 (78.54%) | 0.001 |
| Statins | 192 (98.97%) | 217 (99.09%) | 0.903 |
| Aldosterone-antagonist | 53 (27.32%) | 38 (17.35%) | 0.015 |
| **Clinical outcomes** |  |  |  |
| Recurrent ischemia | 24 (12.12%) | 13 (5.88%) | 0.025 |
| Recurrent MI | 3 (1.52%) | 2 (0.90%) | 0.566 |
| AF or flutter | 14 (7.07%) | 8 (3.62%) | 0.114 |
| HF | 45 (22.73%) | 23 (10.41%) | <.001 |
| Cardiogenic Shock | 11 (5.56%) | 4 (1.81%) | 0.039 |
| VF or VT cardiac arrest | 19 (9.60%) | 10 (4.52%) | 0.041 |
| Stroke | 2 (1.01%) | 0 (0.00%) | 0.134 |
| In-hospital mortality | 4 (2.02%) | 2 (0.90%) | 0.337 |
| One-month mortality | 5 (3.40%) | 4 (2.31%) | 0.557 |
