# Supplementary material for: Acute myocardial infarction and acute heart failure in the Middle East and North Africa: Study design and pilot phase study results from the PEACE MENA registry
Source: PLoS One. 2020 Jul 22;15(7):e0236292. doi: 10.1371/journal.pone.0236292 (PMC7375595; doi:10.1371/journal.pone.0236292)
Supplement: S3 Table — (DOCX) [file pone.0236292.s004.docx]

**S 3 Table.**

**Modalities and timing of reperfusion in the STEMI population.**

|  | **Total**  **N=312** |
| --- | --- |
| **Reperfusion modalities** |  |
| Thrombolysis | 75 (24.04%) |
| Failed thrombolysis | 35 (46.67%) |
| Rescue PCI | 30(9.61%) |
| Pharmaco-Invasive approach | 30(9.61%) |
| Primary PCI | 176 (56.41%) |
| No reperfusion therapy | 61(19.55%) |
| **Reasons for no reperfusion therapy** |  |
| Late presentation | 49 (80.33%) |
| Missed | 4 (6.56%) |
| Contraindication | 2 (3.28%) |
| Other | 6 (9.84%) |
| **Timing of reperfusion** |  |
| Symptom to ER Minutes, Median (IQR) | 255.0 (1200) |
| Door to ECG Minutes, Median (IQR) | 10.00 (10.00) |
| Door to ECG < 10 Minutes | 248 (45.67%) |
| Door to needle Minutes, Median (IQR) | 80.00 (60.00) |
| Door to needle < 30 Minutes | 2 (6.06%) |
| Door to balloon Minutes, Median (IQR) | 63.00 (260.0) |
| Door to balloon < 90 Minutes | 109 (61.93%) |
