# Supplementary material for: Acute myocardial infarction and acute heart failure in the Middle East and North Africa: Study design and pilot phase study results from the PEACE MENA registry
Source: PLoS One. 2020 Jul 22;15(7):e0236292. doi: 10.1371/journal.pone.0236292 (PMC7375595; doi:10.1371/journal.pone.0236292)
Supplement: S4 Table — (DOCX) [file pone.0236292.s005.docx]

**S 4 Table.**

**Educational level and socio-economic status in the AHF population**

| **Education** | **Total** |
| --- | --- |
| None | 82/331 (24.77%) |
| Primary | 92/331 (27.79%) |
| Secondary/High School/Diploma | 84/331 (25.38%) |
| Trade/vocational school | 29/331 (8.76%) |
| College/university | 37/331 (11.18%) |
| Post-graduation degree. e.g. PHD, Master | 7/331 (2.11%) |
| **Average total monthly household income including subsidies** |  |
| < 500 $ | 173/331 (52.27%) |
| 500-2000 $ | 97/331 (29.31%) |
| 2000-4000 $ | 25/331 (7.55%) |
| >4000 $ | 36/331 (10.88%) |
| Number of dependent household members - Median(IQR) | 4.00 (4.00 ) |
| **Difficulties in paying bills or buying food or clothes in the last year** |  |
| All Time | 32/330 (9.70%) |
| often | 42/330 (12.73%) |
| sometimes | 65/330 (19.70%) |
| rarely | 65/330 (19.70%) |
| never | 126/330 (38.18%) |
| **Coverage of medical care expenses** |  |
| Difficulties to afford medical care expenses | 92/152 (60.53%) |
| Coverage by a private medical insurance | 73/339 (21.53%) |
| Coverage by free governmental medical care | 115/267 (43.07%) |
| **Occupation.** |  |
| Self-employed (as Independent, or have own business) | 51/340 (15.00%) |
| Employee (as salesperson, director, accountant) | 49/340 (14.41%) |
| Retired | 107/340 (31.47%) |
| Unemployed (as housewife, househusband) | 133/340 (39.12%) |
