# Supplementary material for: Acute myocardial infarction and acute heart failure in the Middle East and North Africa: Study design and pilot phase study results from the PEACE MENA registry
Source: PLoS One. 2020 Jul 22;15(7):e0236292. doi: 10.1371/journal.pone.0236292 (PMC7375595; doi:10.1371/journal.pone.0236292)
Supplement: S5 Table — (DOCX) [file pone.0236292.s006.docx]

**S 5 Table**

Clinical features, management, and outcomes of patients with lower versus higher income presenting with acute heart failure

|  | lower income (<=500$/month)  n=173(52.27%) | Higher income (>500$/month)  n=158(47.73%) | P-value |
| --- | --- | --- | --- |
| Age (y), mean ±SD | 57.62 ± 17.29 | 63.58 ± 12.72 | <.001 |
| Male | 108 (62.43%) | 105 (66.46%) | 0.445 |
| Type of HF |  |  | <.001 |
| Acute on chronic HF | 90 (52.02%) | 118 (74.68%) |  |
| Acute De novo HF | 83 (47.98%) | 40 (25.32%) |  |
| Residency |  |  | <.001 |
| GCC country (KSA, Kuwait, Qatar, Bahrain, UAE, Oman) | 21 (12.14%) | 53 (33.54%) |  |
| Non-GCC country (all other countries together) | 152 (87.86%) | 105 (66.46%) |  |
| Low education (none, primary, secondary) | 140 (80.92%) | 118 (74.68%) | 0.171 |
| **Main Cause of HF** |  |  | 0.039 |
| Ischemic | 73(42.20%) | 92(58.23%) |  |
| Non-ischemic (any other cause) | 100(57.80%) | 66(41.77%) |  |
| **Medical history** |  |  |  |
| HTN | 98 (56.65%) | 105 (66.46%) | 0.067 |
| Diabetes | 77 (44.51%) | 88 (55.70%) | 0.042 |
| Current or ex-smoker | 39 (22.54%) | 34 (21.52%) | 0.822 |
| Dyslipidemia | 50 (28.90%) | 63 (39.87%) | 0.035 |
| Prior angina or MI | 73 (42.20%) | 96 (60.76%) | <.001 |
| Prior PCI | 27 (36.99%) | 33 (34.38%) | 0.725 |
| Prior CABG | 14 (19.18%) | 15 (15.63%) | 0.544 |
| Heart Failure | 90 (52.02%) | 118 (74.68%) | <.001 |
| Stroke | 15 (8.67%) | 16 (10.13%) | 0.650 |
| Chronic kidney disease | 35 (20.23%) | 41 (25.95%) | 0.217 |
| **Clinical presentation** |  |  |  |
| HR>100 bpm | 75 (43.35%) | 42 (26.58%) | 0.001 |
| BP< 90 mmHg | 21 (12.14%) | 7 (4.43%) | 0.012 |
| **Cardiac Procedures** |  |  |  |
| Echo: Moderate or severe LV dysfunction | 84 (50.60%) | 91 (58.71%) | 0.145 |
| PCI | 8 (4.62%) | 9 (5.70%) | 0.659 |
| CABG | 0 (0.00%) | 1 (0.63%) | 0.295 |
| **Medications at discharge** |  |  |  |
| Anti-platelets (Aspirin, Clopidogrel, Ticagrelor) | 96 (55.49%) | 102 (64.56%) | 0.092 |
| Beta-blockers | 127 (73.41%) | 115 (72.78%) | 0.898 |
| ACE-Is/ARBs | 113(65.32%) | 79(50.00%) | 0.004 |
| Statins | 96 (55.49%) | 98 (62.03%) | 0.228 |
| Aldosterone-antagonist | 91 (52.60%) | 86 (54.43%) | 0.738 |
| Furosemide | 152(87.86%) | 145(91.77%) | 0.241 |
| **Clinical outcomes** |  |  |  |
| Intubation Ventilation | 19 (10.98%) | 7 (4.43%) | 0.027 |
| IABP | 2 (1.16%) | 1 (0.63%) | 0.616 |
| Acute Dialysis Ultrafiltration | 4 (2.31%) | 4 (2.53%) | 0.897 |
| VT/VF Requiring Therapy | 14 (8.09%) | 9 (5.70%) | 0.392 |
| AF Requiring Therapy | 22 (12.72%) | 17 (10.76%) | 0.581 |
| Major bleeding | 2 (1.16%) | 2 (1.27%) | 0.927 |
| Blood Transfusion | 10 (5.78%) | 2 (1.27%) | 0.028 |
| Stroke | 2 (1.16%) | 1 (0.63%) | 0.616 |
| In-hospital mortality | 10 (5.95%) | 7 (4.58%) | 0.582 |
| One-month mortality | 15 (9.32%) | 7 (4.83%) | 0.129 |
